# Genomic insights into adaptative traits of phyllosphere yeasts

**DOI:** 10.1186/s40793-025-00839-7

**Published:** 2026-01-03

**Authors:** Linda Gouka, Cristina Serra i Melendez, Nelli Vardazaryan, Knud Nor Nielsen, Leise Riber, Lars Hestbjerg Hansen, Jos M. Raaijmakers, Michael F. Seidl, Chrats Melkonian, Viviane Cordovez

**Affiliations:** 1 Department of Microbial Ecology, Netherlands Institute of Ecology (NIOO-KNAW), Wageningen, The Netherlands; 2 Armenian Bioinformatics Institute, Yerevan, Armenia; 3 Institute of Molecular Biology, National Academy of Sciences of Armenia, Yerevan, Armenia; 4 DTU Biosustain, Lyngby, Denmark; 5 Department of Plant and Environmental Sciences, University of Copenhagen, Copenhagen, Denmark; 6 Department of Molecular Biotechnology, Institute of Biology, Leiden, The Netherlands; 7 Department of Theoretical Biology & Bioinformatics, Utrecht University, Utrecht, The Netherlands; 8 Bioinformatics Group, Wageningen University and Research, Wageningen, The Netherlands

## Abstract

**Background:**

Yeasts are ubiquitous microorganisms thriving in diverse environments. They are prevalent members of the phyllosphere microbiome, but genomic studies of plant-associated yeasts remain limited.

**Results:**

We established a taxonomically diverse yeast culture collection from flag leaves of field-grown wheat. This collection captured between 48-56% of the genus-level diversity detected by ITS amplicon sequencing conducted over two consecutive years, including the core members *Aureobasidium*, *Dioszegia*, *Filobasidium*, *Papiliotrema*, *Sporobolomyces*, and *Vishniacozyma*. De novo sequencing of 96 high-quality genomes from this collection, representing 14 yeast genera, and comparative genomics revealed specific signatures associated with life in the phyllosphere, the aboveground part of the plant. These adaptive traits encompass enriched carbohydrate metabolism, secondary metabolite biosynthetic pathways, and pectin degradation. The substantially smaller genomes of the phyllosphere yeast genera *Candida* and *Metschnikowia* suggest niche specialization via prioritizing metabolic pathways that are essential for survival in the nutrient-limited phyllosphere.

**Conclusions:**

This study represents a significant advancement in our understanding of the diverse and largely unknown genomic traits of environmental yeasts and their adaptation to life in the phyllosphere environment. Our findings highlight their untapped functional potential for biotechnological applications in sustainable crop production.

**Supplementary Information:**

The online version contains supplementary material available at https://doi.org/10.1186/s40793-025-00839-7.

## Background

Yeasts are unicellular eukaryotic microorganisms within the fungal kingdom comprising several unrelated lineages [1]. Ascomycota yeasts, also known as ‘true yeasts’ or ‘fission yeasts’, are typically divided by budding or binary fission. In contrast, Basidiomycota yeasts often include dimorphic filamentous fungi, alternating between yeast-like and filamentous growth stages. Approximately 1500 yeast species have been described to date and are estimated to make up 1% of all fungal species [1, 2]. Among these, *Saccharomyces cerevisiae*, known for its roles in fermentation of beer, wine and bread, and *Candida albicans,* an opportunistic human pathogen, hold significant economic, medical, and scientific importance. Beyond these two examples, yeasts serve diverse biotechnological applications, including a largely untapped resource for novel enzymes [3], biocontrol agents of postharvest diseases [4], probiotics [5], and as food and dietary supplements [6].

Yeasts thrive in a wide range of environments, ranging from soil, water, plants, and air to extreme conditions in the Antarctic, hot springs, deep seas, and desserts [7]. The diversity of biotic and abiotic stresses encountered in these environments drives diversification and evolution [8]. One notable example of adaptation is the long-term co-evolution of plants and their microbiota over at least 400 million years, exemplified by the intricate symbiosis between arbuscular mycorrhiza and plants in 70–90% of land plant species [9]. Plant–microbe interactions encompass a spectrum of symbiotic relationships (*e.g.* mutualistic, commensal, and pathogenic) that can influence nutrient uptake, phytohormone production, and stress tolerance, impacting plant growth and health [10]. Research on the phyllosphere, the aboveground parts of plants, has primarily focused on plant pathogens, while the role of beneficial yeasts and their adaptation to the harsh conditions in the phyllosphere (*e.g.* UV radiation and nutrient limitation) remains largely unknown. Additionally, the availability of whole genome sequences from environmental yeasts not linked to industrial applications or medical importance remains scarce.

Genomic studies are starting to shed light on genetic traits that facilitate successful microbial colonization of plant environments. To date, these studies have focused primarily on bacteria [11–14]. Closely related bacterial species or strains exhibit substantial genomic diversity due to niche-driven divergence, aiding in the adaptation to specialized lifestyles or environments [14]. For instance, Levy et al. (2018) employed a computational approach to identify plant-associated bacterial genes. Their findings revealed that plant-associated bacteria are enriched in carbohydrate metabolic functions compared to related non-plant-associated bacteria, along with fewer mobile genetic elements [12]. Similarly, Bai et al. (2015) studied *Arabidopsis* leaf and root isolates, identifying overlaps in taxonomic composition and functional categories. They found that 48–58% of isolates shared ≥ 97% sequence identity with corresponding 16S rRNA genes between the rhizosphere and phyllosphere, suggesting that many taxonomic lineages can colonize both roots and leaves. However, niche specialization was also evident [15]. Genomes of leaf isolates exhibited an enrichment of carbohydrate metabolic genes, presumably due to the presence of complex organic carbohydrates (*e.g.* polysaccharides) on leaves, as opposed to the chemically ‘simpler’ root exudates available in the rhizosphere [15]. Additionally, biotrophic plant pathogens were reported to contain fewer biosynthetic gene clusters and Carbohydrate-Active Enzymes (CAZymes), involved in plant cell wall degradation, compared to necrotrophs and hemibiotrophs, which highlights their adaptation to live plant tissue [16]. Despite these advances, most research to date has not focused on plant-associated yeasts, partly due to the limited availability of genomes of environmental yeasts.

Currently, the number of available yeast genomes is far lower than that of bacteria and filamentous fungi, especially those originating from the phyllosphere environment. Specific genomic features have been hypothesized to be associated with successful colonization of particular environments. For instance, ACC deaminase production by phyllosphere symbionts has been shown to promote plant growth [17], the gene count of fungal lactamases has been linked to environmental complexity (*e.g.* microbial composition and competition) [18], CAZymes enable nutrient acquisition in poor environments [19], and secondary metabolite production contributes to defense and signaling [20]. Hence, comparative genomics approaches allow insights into adaptations of yeasts to live in close association with their host plants and to disentangle the underlying molecular and chemical mechanisms of host adaptation.

In this study, we profiled the fungal community of the flag leaves of field-grown wheat to investigate the diversity and dynamics of yeasts and pathogenic filamentous fungi. We integrated ITS metabarcoding data with our unique yeast culture collection to compare the taxonomic diversity based on culture-independent and –dependent approaches, respectively. Additionally, we performed large-scale genome sequencing and computational analysis of 96 environmental yeasts inhabiting the wheat phyllosphere. Our findings reveal large genomic diversity of yeasts and highlight key adaptative traits for the phyllosphere environment, including enriched carbohydrate metabolism, secondary metabolite pathways, and pectin degradation. This study represents a significant advancement in our understanding of the diverse and unknown functional traits of environmental yeasts and their adaptation to life in the harsh phyllosphere environment.

## Results

### Yeast community composition of wheat flag leaves

To assess the yeast composition of field-grown wheat over two consecutive years of field trials (2020 and 2021), we employed community profiling by Internal Transcribed Spacer (ITS) rRNA gene sequencing (Supplementary Table 1). A total of 339 fungal genera were identified, including 43 yeast and 29 yeast-like genera (hereafter referred to as yeasts) accounting for the majority of reads, with a total relative abundance of 61,8% (SD ± 1,7%) in 2020, and 46,2% (SD ± 1,6%) in 2021. After excluding low abundance reads (relative abundance < 0.1% in both years), 23 and 18 yeast genera (Supplementary Table 2) remained, respectively. Pathogenic yeasts belonging to *Ustilago*, *Malassezia*, and *Itersonilia* accounted for more than half of the yeast community in 2020 (36,2% relative abundance to total ITS reads), and a reduced proportion in 2021 (15,9% relative abundance to total ITS reads), even though leaves did not show visible signs of pathogen infections.

**Fig. 1:**
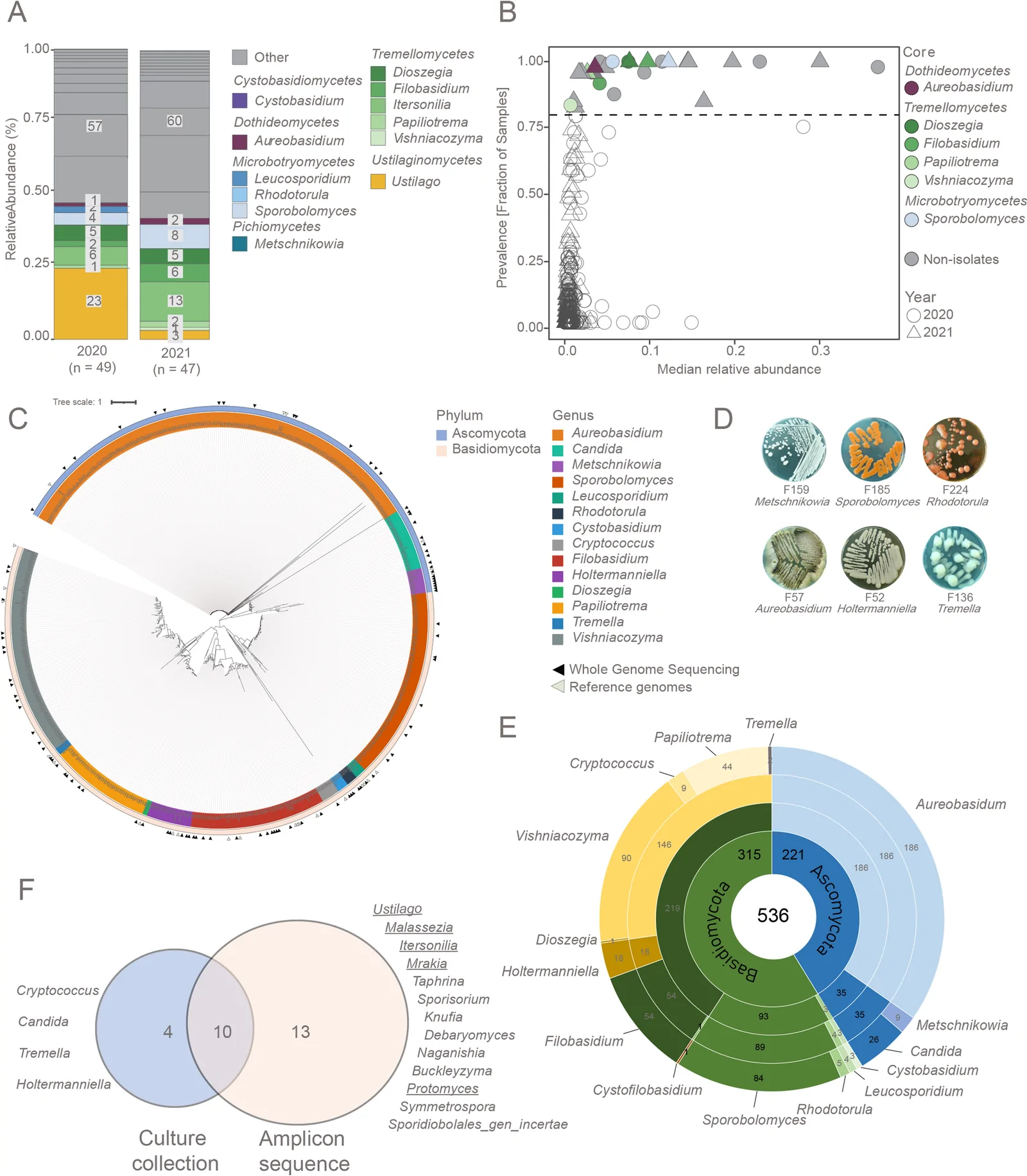
Taxonomic diversity of yeasts from flag leaves of wheat over two consecutive field seasons. Panels A and B include amplicon sequence variants (ASVs) assigned as yeast, yeast-like and pathogenic filamentous fungi, whereas Panels C-F focus on yeast and yeast-like isolates. **(A)** General distribution of the phyllosphere yeast and pathogenic fungi community over two years based on ITS amplicon sequencing. Numbers in the bar plots indicate the number ASVs of per genera. All ASVs which did not belong to yeast, yeast-like, or pathogenic fungi, or those with a relative abundance < 1.0% were grouped into the category ‘Other’. **(B)** Prevalence of yeast genera per field season. Colored shapes indicate the core mycobiome (i.e. present in at least 80% of wheat flag leaves per year). **(C)** Phylogenetic tree of the yeast isolates based on ITS amplicon sequences compared to reference yeast sequences in the NCBI database. Reference yeast sequences are represented by their accession number and indicated with grey triangles), while yeast isolates from the collection are denoted by an “F” followed by a number (listed in Supplementary Table 3). Order of the isolates in the tree follows the same order as presented in the legend on the right. The yeast isolates selected for whole genome sequencing (based on their taxonomic rank and abundance in the culture collection) are indicated by a black triangle. **(D)** Phenotypic characteristics of 6 yeast genera on PDA after 7-10 days of growth at 25°C. **(E)** The number of yeast isolates per genus, order, class and, phylum. The total number of isolates is indicated in the center, and per taxonomic rank. **(F)** Venn diagram displaying the shared and unique yeast and yeast-like taxa in the culture collection and in the ITS amplicon sequencing data

Taxonomic diversity was significantly higher in 2021, even though the number of observed ASVs remained constant (Supplementary Fig. 1A; Wilcoxon test, p < 0.05). Differential abundance analysis using ANCOM-BC2 revealed significant increases in abundance of taxa belonging to the yeast genera *Vishniacozyma*, *Sporobolomyces*, *Papiliotrema*, and *Aureobasidium* in 2021 (Supplementary Fig. 1B). These taxa exhibited high prevalence across samples with varying median relative abundances. The consistent presence of abundant genera across both years suggests the existence of a stable core mycobiome (Fig. 1A, B), which was present in at least 80% of samples per year. More specifically, the core yeast community included *Aureobasidium*, *Dioszegia*, *Filobasidium*, *Papiliotrema*, *Sporobolomyces*, and *Vishniacozyma*. Notably, *Papiliotrema* and *Aureobasidium* were core members only in 2021, suggesting environmental or host-related factors influence their persistence.

In parallel, we also established a culture collection of yeasts from the wheat flag leaves harvested in 2020 and 2021. Sanger sequencing of the ITS region was performed for taxonomic delineation of 536 yeast isolates. Recovery estimates of the culture collection compared to leaf-associated amplicon sequence variants (ASVs) varied over the two years between 47,8 to 55,6% at a relative abundance of ≥ 0,1%. These isolates represented 14 different genera belonging to Ascomycota (41%) and Basidiomycota (59%) (Fig. 1C, D). The highly conserved ITS regions found in some yeast species resulted in insufficient genetic variation to reliably distinguish between closely related species. The culturable fraction of the community isolated on different agar media was dominated by *Aureobasidium* (186). *Vishniacozyma* (90), *Filobasidium* (55), and *Sporobolomyces* (84) were also well-represented, while other genera, such as *Dioszegia* and *Tremella* were rare, with only one or two isolates (Fig. 1E). Out of the 14 genera detected in the culture collection, 10 were also detected in the amplicon sequencing dataset, including *Aureobasidium, Metschnikowia, Cystobasidium, Dioszegia, Filobasidium, Leucosporidium, Papiliotrema, Rhodotorula, Sporobolomyces,* and *Vishniacozyma* (Fig. 1F, Supplementary Fig. 2). Among these, *Dioszegia, Filobasidium, Aureobasidium* and *Sporobolomyces* had a relative abundance greater than 1% in both sampling years. Out of the 23 genera detected in community profiling (mean relative abundance ≥ 0,1%), 13 were not present in the culture collection, including five pathogens (*Ustilago*, *Malassezia*, *Itersonilia*, *Mrakia*, and *Protomyces*). Besides, 17 genera were overlapping between the two consecutive amplicon sequencing years, whereas six genera were solely detected in 2020 (*Rhodotorula*, *Debaryomyces*, *Naganishia*, *Knufia*, *Protomyces*, and *Symmetrospora*), while only one genus (*Buckleyzyma*) was detected only in 2021.

### Expanding the genome catalogue of phyllosphere yeasts

To expand the genome catalogue and to explore the functional repertoire of yeasts from phyllosphere environments, 96 isolates were selected based on taxonomic diversity and abundance in the culture collection (Fig. 1C, Supplementary Table 4, Supplementary Fig. 3). More specifically, we selected at least two representatives for each genus (when available), and the genera with a higher relative abundance in the culture collection were represented by more isolates in the genome collection. The selected 96 yeast isolates covered 14 genera, including *Aureobasidium* (n = 14), *Candida* (n = 8), *Cystobasidium* (n = 3), *Dioszegia* (n = 3), *Filobasidium* (n = 16), *Holtermanniella* (n = 6), *Leucosporidium* (n = 1), *Metschnikowia* (n = 10), *Papiliotrema* (n = 7), *Pseudohyphozyma* (n = 1), *Pseudotremella* (n = 2), *Rhodotorula* (n = 3), *Sporobolomyces* (n = 7), and *Vishniacozyma* (n = 15).

**Fig. 2:**
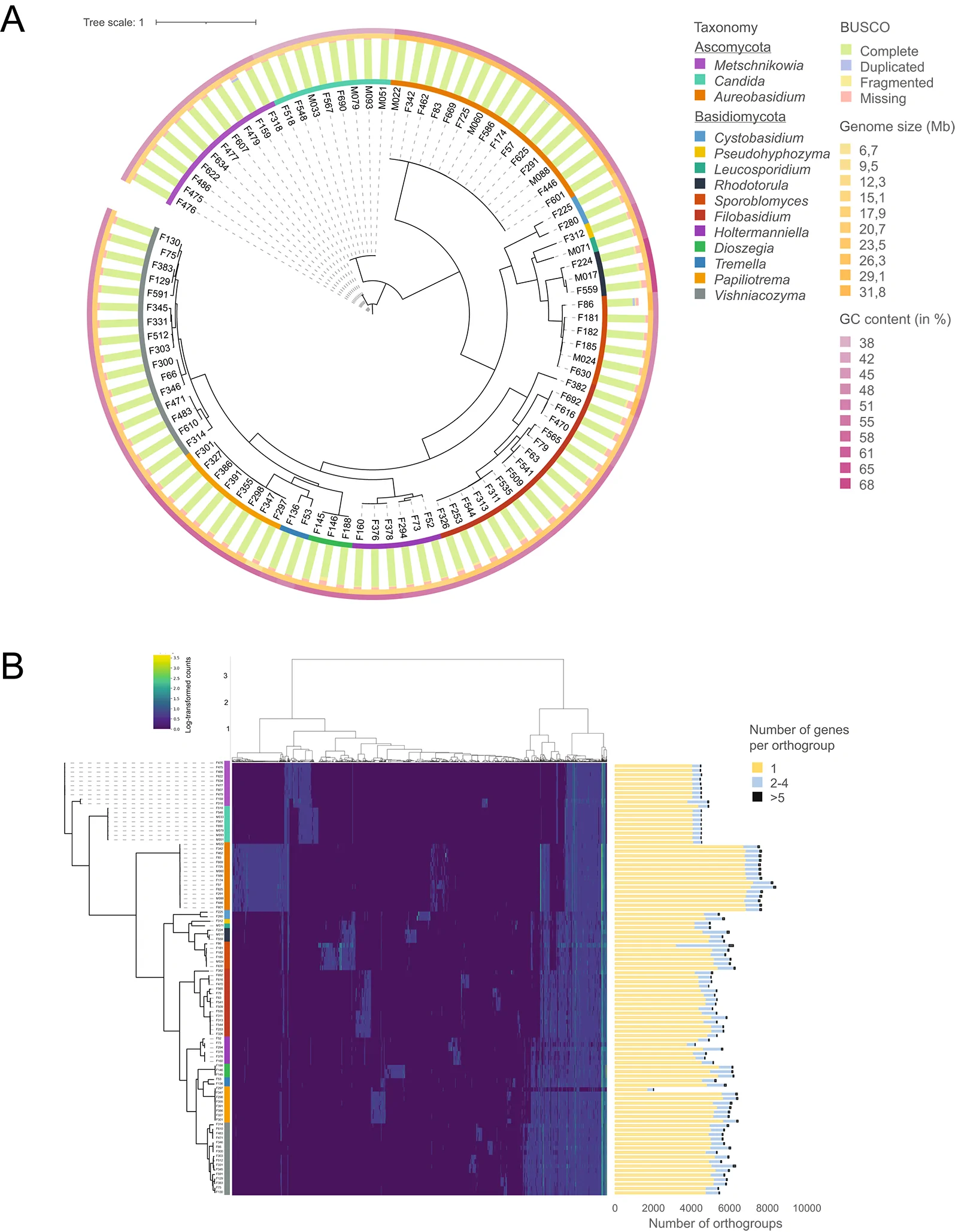
Phylogenetic tree and inter-and intra-species variation of 96 wheat phyllosphere yeasts. (**A**) Maximum likelihood phylogeny based on 758 BUSCO single-copy genes of the whole yeast genomes. Genome statistics are shown for BUSCO, genome size (in Mb; orange), and GC content (in %; purple). Color stripes highlight low to higher values, ranging from light to dark color codes. (**B**) Heatmap of clustered log-transformed orthogroup gene counts. Yeast genera color coded based on taxonomy follows the same order as presented in the taxonomy legend. Stacked bar plot (right) indicates the number of orthogroups with gene counts

To assess genome assembly completeness and phylogenetic relationship, a total of 758 Benchmarking Universal Single-Copy Orthologs (BUSCO) genes were used (Fig. 2A). On average, 693 BUSCO genes were identified per genome, with an average BUSCO completeness of 92%. *Aureobasidium* had the most complete genome assemblies, ranging from 743 to 751 out of 758 BUSCO genes present, followed by *Candida* with 731 to 734 genes. In contrast, 478 and 603 BUSCO genes were complete for *Sporobolomyces* sp. F86 and *Dioszegia* sp. F146, probably due to the high number of contigs (1,344 and 2,413, respectively), indicating a less complete assembly and more fragmentation. The average genome size of the 96 newly sequenced phyllosphere yeasts was 20.8 Mb, ranging from 31.8 Mb (*Sporobolomyces* sp. F86) to 6.7 Mb (*Papiliotrema* sp. F297). The largest average genome sizes per genus were found for *Aureobasidium* (29.2 Mb), followed by *Sporobolomyces* (23.2 Mb) and *Cystobasidium* (22.2 Mb), while the smallest genomes were observed for *Holtermanniella* (18.5 Mb), *Metschnikowia* (16.0 Mb) and *Candida* (14.1 Mb) isolates. Phyllosphere yeast genomes displayed 51% GC content on average. *Rhodotorula* and *Papiliotrema* isolates showed the highest GC content (68% and 58%, respectively), whereas *Candida* and *Metschnikowia* isolates showed the lowest GC content (38% and 45.5%, respectively).

Comparative genomics by orthogroup analysis (*i.e.* groups of genes that are orthologous) of the 14 yeast genera (Fig. 2B) revealed a total of 690,814 genes of which 682,566 genes (98.8%) were assigned to 25,903 orthogroups, with a mean orthogroup size of 26.4 (gene count). *Aureobasidium* contained most unique orthogroups (4201), followed by *Filobasidium* (2,258) and *Vishniacozyma* (2,057), indicating large interspecific genome diversity. Most overlap between genera was observed for *Candida* and *Metschnikowia* (907 orthogroups), followed by *Sporobolomyces*, *Rhodotorula* and *Leucosporidium* (397 orthogroups), suggesting genomic similarities between these genera.

To investigate the differences in functional traits across the culture collection, we used COG (Cluster of Orthologous Groups) functional category enrichment analysis to annotate the unique and shared orthogroups per functional category across genera. Expected COG values were calculated based on the soft core (95%; based on 1966 orthogroups) and then compared to the observed outcome per functional category and genera (Fisher’s test and Benjamini–Hochberg correction, p < 0.05) (Supplementary Fig. 4). Despite their small genome sizes, *Candida* and *Metschnikowia* displayed significant functional specialization (> 50%) with enrichment in eight and four out of 23 COG functional categories, respectively. Genes associated with secondary metabolism were overrepresented in unique (*Metschnikowia* (92%) and *Candida* (215%)), and shared (145%) orthogroups, and those shared among *Candida*, *Metschnikowia* and *Aureobasidium* (93%). Defense-related genes were also enriched in these genera (*Candida*, *Metschnikowia* and *Aureobasidium* (716%)). Cell wall and membrane functions were overrepresented in multiple genera except *Leucosporidium* (103%), and underrepresented in *Aureobasidium* (51%), *Rhodotorula* (57%) and *Cystobasidium* (51%). Lastly, carbohydrate metabolism genes were enriched in the unique orthogroups of *Candida* (89%) and those shared with *Metschnikowia* (118%), while underrepresented in *Aureobasidium* (52%), suggesting functional differentiation between phyllosphere-associated yeasts.

Secondary metabolites are known to play an important role in colonization and persistence. Therefore, we mined the new yeast genomes for biosynthetic gene clusters (BGCs) using fungiSMASH, and identified 824 BGCs, including 335 non-ribosomal peptide synthetases (NRPS)-like, 249 terpenes and 72 T1-polyketide synthases (T1PKS) (Supplementary Fig. 5). *Aureobasidium* genomes exhibited the highest BGC number and diversity (21–31 BGCs), including T1 polyketide synthases (T1PKS), fungal-RiPP-like proteins, NRPS-T1PKS hybrids, NRPS with β-lactone activity, and a NRP-metallophore. In contrast, only two BGCs were identified for *Metschnikowia* and *Candida*, a terpene and a NRPS-like cluster, despite their functional enrichment in secondary metabolites. FungiSMASH identified β-lactone BGCs in the genomes of *Aureobasidium*, *Rhodotorula* and *Sporobolomyces* genomes, while *Aureobasidium* and *Papiliotrema* harbored BGCs for non-alpha poly-amino acids (NAPAA). Only eleven BGCs displayed amino acid (aa) similarities (12%—100%) with known secondary metabolite clusters, including genes for the terpene clavaric acid (a farnesyltransferase inhibitor; 39 isolates, aa 100% similarity) and choline (structural component of phospholipids and cell membranes; 50 isolates, aa 100% similarity). Other BGCs which could be annotated for *Aureobasidium* genomes included: chaetoglobosin A/C (anticancer, 57%—100% aa similarity), burnettramic acid A (antimicrobial, 22%—33% aa similarity), the siderophore metachelin A/C/A-CE/B (dimerumic acid, antioxidant, 25% aa similarity), monascorubrin (antibacterial, 100% aa similarity), scytalidin (fungitoxic, 12% aa similarity), scytalone, (melanin precursor, 40% aa similarity) and clusters with yet unknown functions such as 1,3,5,8-tetrahydroxynapthalene (100% aa similarity) and UNII-YC2Q1094PT (100% similarity). Interestingly, a hit for the sesquiterpenoid phytohormone abscisic acid (ABA) was identified for two *Aureobasidium* isolates (50% aa similarity). Collectively, these results give new insights into the functional diversity of phyllosphere yeasts, but also indicate that fungiSMASH is limited in detecting unknown gene clusters in many of these yeast species. To improve predictions of the functional potential of environmental yeasts, further studies should expand the catalog of their exometabolome.

To assess colonization potential of yeasts in the leaf phyllosphere, we analyzed their enzymes involved in biosynthesis, modification, and degradation of complex carbohydrates using the CAZyme database. Across the 96 yeast genomes, we identified 78 distinct CAZyme families, with glycoside hydrolases (GH, 29 classes) and glycosyltransferases (GT, 41 classes) as the most abundant (Supplementary Fig. 6). Each genome encoded on average 130 CAZymes, with large variability among yeast isolates. *Papiliotrema* sp. F355 had the highest number with 213 CAZymes, followed by the 15 *Aureobasidium* isolates with 190 to 199 CAZymes. Genera with the largest genomes, like *Aureobasidium*, showed the highest average CAZyme count (194), while *Candida* and *Metschnikowia* had much less CAZymes (98 CAZymes on average). To compare CAZyme frequency independent of genome size, we normalized the CAZyme counts per megabase (CAZymes/Mbp). Using this normalization, *Papiliotrema* sp. F355 showed the highest number of CAZymes (9.7 on average), whereas *Sporobolomyces* (4.5), *Rhodotorula* (4.8) and *Cystobasidium* (4.8) showed the lowest frequency. This variation in CAZyme frequency may reflect different levels of specialization and resource use among the yeast genera and isolates. Higher CAZyme densities may provide adaptation to the nutrient-poor phyllosphere environment to breakdown complex plant substrates, while fewer CAZymes may provide more efficient metabolization of more readily available substrates in phyllosphere niches where intense competition for complex carbohydrates is less critical.

In summary, our analysis revealed distinct genomic and functional diversification among phyllosphere yeasts. Despite their smaller genome sizes, *Candida* and *Metschnikowia* isolates displayed significant enrichment in several functional categories, including secondary metabolism and defense mechanisms. *Aureobasidium* isolates exhibited the highest biosynthetic gene cluster (BGC) diversity, predicted to encode compounds with antimicrobial and antioxidant activity. However, fungiSMASH was limited in detecting other putative secondary metabolite pathways in *Candida* and *Metschnikowia* isolates. CAZyme profiling revealed substantial variation among the 96 yeast genomes and genera, with *Aureobasidium* and *Papiliotrema* isolates having the highest CAZyme counts, potentially aiding in plant polysaccharide degradation.

### Cataloguing known yeast genomes from different environments

**Fig. 3:**
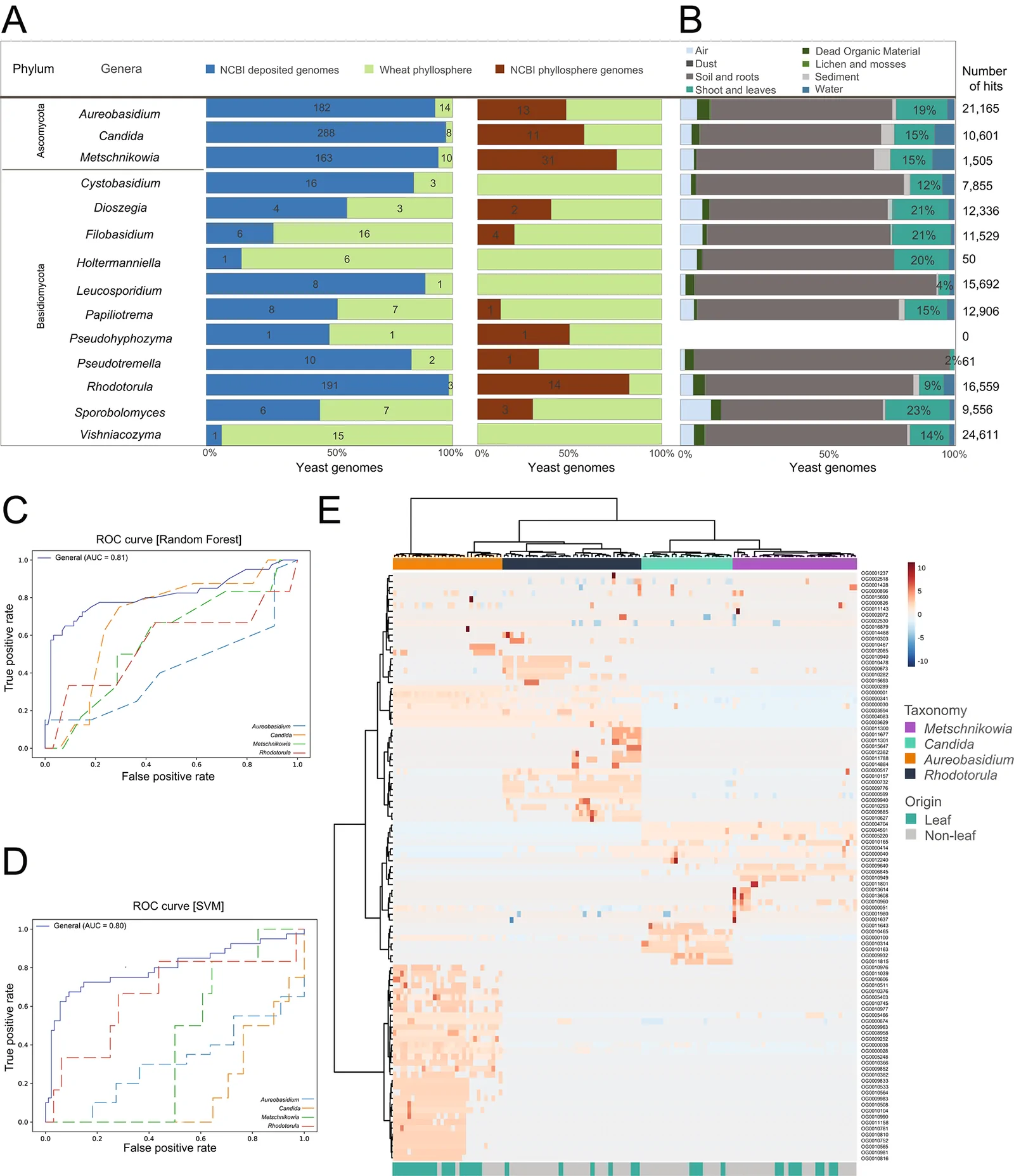
Yeast genomic data from various origin sources. (**A**) 14 yeast genera detected in the wheat phyllosphere. The first stacked bar plot shows the number of all available yeast genomes in the NCBI database (in blue) and those detected in the wheat phyllosphere (this study). The second stacked bar plot shows the number of NCBI genomes exclusively of phyllosphere origin (*e.g.* flowers and leaves; in brown) and the newly sequenced genomes from wheat phyllosphere yeasts (in green). (**B**) Yeast origin based on the sequence variants deposited at the GlobalFungi database. Origins were consolidated into 8 categories, and the number of sequence hits per genus is indicated at the end of each bar. No information was available for *Pseudohyphozyma*. The percentage of shoot and leaf-associated yeasts are represented in the corresponding bar (color-coded based on origin). (**C, D**) Receiver Operating Characteristic curve for genomes with leaf vs non-leaf genomic origin based on (**C**) Random Forest, and (**D**) Support vector machine indicating the false positive vs true positive rates. The dark blue solid line indicates the overall model performance (AUC), while the dashed lines indicate the LOGO model performances for the four taxonomic groups when each was left out as the testing set. (**E**) Heatmap of the top 100 important features in the random forest model. Isolates are color coded based on genera (*Aureobasidium*; orange; *Rhodotorula*; black; *Candida*; light-green; and *Metschnikowia*; purple), and origin (leaf, green; non-leaf, grey)

To explore the genomic diversity and identify leaf-associated traits in our phyllosphere yeast collection, we reviewed the available genomes in the NCBI database for taxonomically related yeasts from other environments (similarity threshold of 99,0%) (Fig. 3A). We classified these publicly available genomes based on their origin of isolation, which included phyllosphere (*i.e.* leaf and flowers) and non-phyllosphere isolates (i.e*.* all other origins). Ascomycete yeasts such as *Aureobasidium* (182 genomes), *Candida* (288 genomes), *Metschnikowia* (163 genomes) and the basidiomycete *Rhodotorula* (191 genomes) were well-represented in the database, although only a small fraction of the database genomes originated from phyllosphere yeasts. All other yeast genera were represented by fewer than 20 genomes, with eight genera having less than ten publicly available genomes. In comparison, members of the *Saccharomyces* genus are represented by approximately 2,000 genomes (including 1,662 *Saccharomyces cerevisiae* genomes), highlighting the fundamental knowledge gap of the genomic diversity of phyllosphere and ‘non-conventional’ yeasts (NCBI genome database, April 2024). By adding our 96 phyllosphere yeast genome sequences into the NCBI database to the existing yeast genomes, the number of *Aureobasidium*, *Candida* and *Metschnikowia* genomes from phyllosphere environments doubled. Moreover, genomes of four genera from the phyllosphere, *Cystobasidium*, *Holtermanniella*, *Leucosporidium*, and *Vishniacozyma*, are represented for the first time (Fig. 3A).

We next surveyed the GlobalFungi database [21] to assess the distribution of the 14 selected yeast genera across different environments (soil, water, plants, and air). All 14 genera were found in the database except for *Pseudohyphozyma*. The available metadata indicated that the selected yeast genera were originally isolated from 17 different environments, which we consolidated into eight categories: dead organic material (*e.g.* dead wood and litter), dust, lichens and mosses, soil and roots (*e.g.* root, rhizosphere soil, soil and topsoil), sediment, shoot and leaves, and water. Environments such as coral, glacial ice debris and fungal sporocarp were excluded due to the very few hits (< 20). *Vishniacozyma* appeared 24,611 times in the database, followed by *Aureobasidium* (21,169) and *Rhodotorula* (16,565) (Fig. 3B). In contrast, *Holtermanniella* and *Pseudotremella* were reported in only 50 and 61 samples, respectively. On average, 15% of the database hits were for yeasts originating from shoot and leaves, with high occurrences for *Sporobolomyces* (23%), *Filobasidium* (21%), *Dioszegia* (21%), *Holtermanniella* (20%), and *Aureobasidium* (19%). These findings indicate that the vast majority of yeast genomic data is derived from soil and rhizosphere environments, likely due to an overrepresentation of soil- and rhizosphere microbiome studies in contemporary databases.

To identify genomic signatures (*e.g.* functional traits or gene groups) of phyllosphere yeasts, we compiled a list of genomes from our yeast culture collection, focusing only on yeast genera with at least 15 available genomes representing different environments, to obtain sufficient computational resolution. Four yeast genera (*Aureobasidium*, *Candida*, *Metschnikowia* and *Rhodotorula*) met these criteria with a total of 859 genomes, with the isolation origin described for 679 genomes only, which were taken for further analysis. After stringent quality control, dereplication (≤ 99%), and balancing the number of genomes from each environmental origin, a final dataset with 128 high-quality genomes was selected for comparison (Supplementary Table 5). These included yeast isolates from leaf (40), flower (26), soil (21), human (23), and water (18).

To determine whether genomic features could reliably predict isolation origin of these yeasts, we trained a machine learning model (Random Forest (RF) and Support Vector Machine (SVM)) using genomic signatures to classify genomes as leaf-associated or non-leaf-associated. The reference model, including the four genera with a high number of genome representatives in both the training and test sets, achieved moderate high accuracy (RF; 0.84 ± 0.07, SVM; 0.82 ± 0.06) and AUC (RF; 0.81, SVM; 0.80). To assess the generalizability of environmental classification across genera, we implemented a Leave-One-Group-Out (LOGO) cross-validation approach, in which the models were trained on three genera and tested on the excluded genus (Supplementary Table 6). To assess classifiers performance in distinguishing leaf-associated from non-leaf-associated genomes, we calculated the accuracies, operating characteristic (ROC) curves for the reference and each LOGO model (Fig. 3C, D, Supplementary Table 7). The mean LOGO classification accuracy was 0.675 ± 0.195 for the RF model and 0.542 ± 0.189 for the SVM model. Similar drop was observed in AUC metric (Fig. 3C-D, Supplementary Table 7). However, model performance varied depending on the excluded genus. The highest classification accuracies were observed when *Rhodotorula* was left out (RF; 0.8421, SVM; 0.8421), followed by *Metschnikowia* (RF; 0.8235, SVM; 0.4118) and *Candida* (RF; 0.68, SVM; 0.56). In contrast, when *Aureobasidium* was excluded, classification accuracy dropped significantly (RF: 0.3549, SVM: 0.3548), indicating that models trained on *Candida*, *Metschnikowia*, and *Rhodotorula* failed to generalize to *Aureobasidium* isolates. Additionally, the highest number of unique orthogroups could be observed for *Aureobasidium* isolates (Fig. 3E). Based on these machine learning results, we applied a genus-specific approach to identify leaf-associated traits, rather than relying on a cross-genus universal model.

### Genomic signatures of phyllosphere-adaptive traits in yeasts

**Fig. 4:**
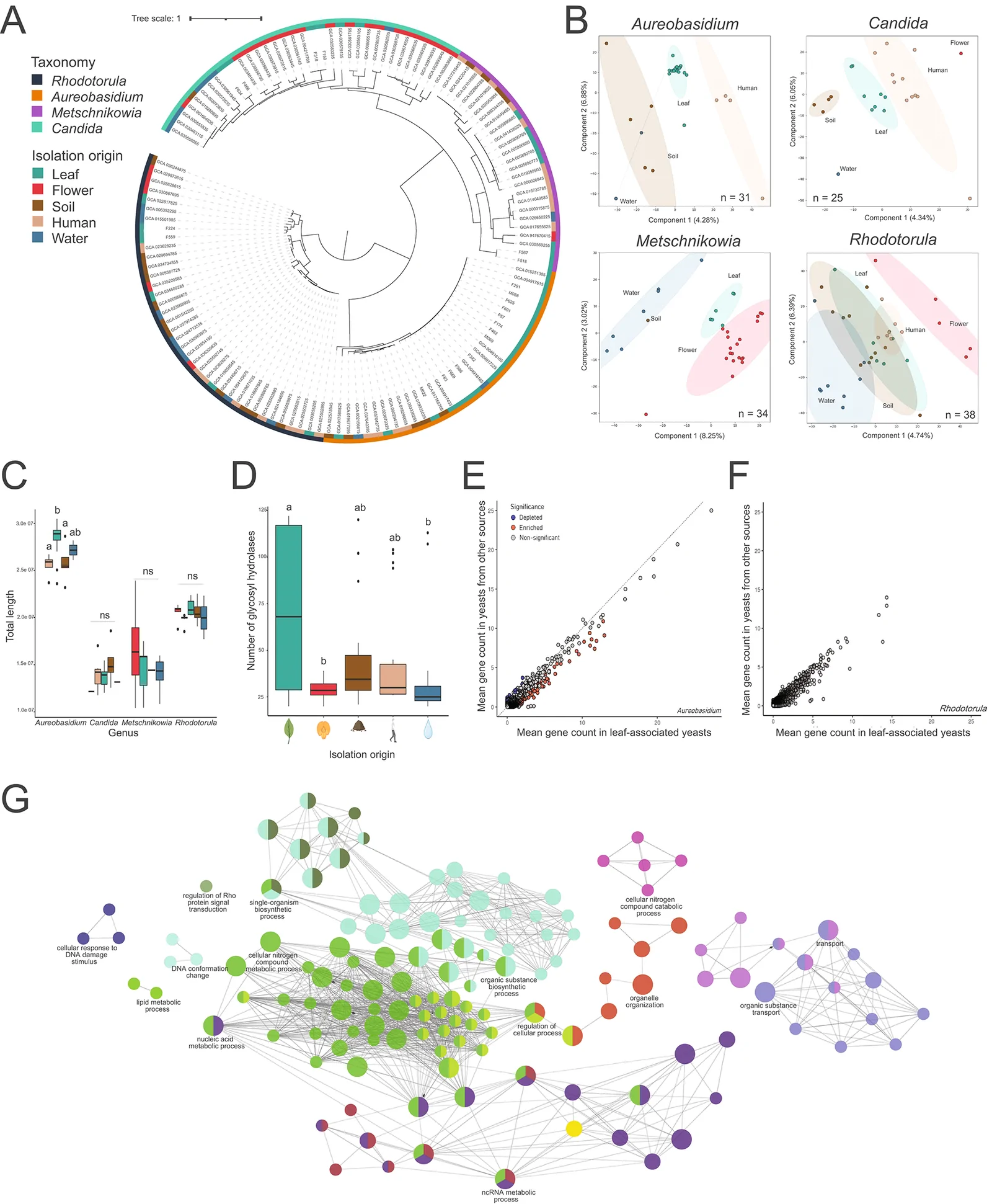
Phylogeny and environmental distribution of yeasts. (**A**) Maximum likelihood tree of 128 yeast high-quality genomes used in the data analysis, based on 758 BUSCO single-copy genes. The outer ring represents taxonomic classification, while the inner ring indicates the isolation source, categorized as human, soil, flower, water and leaf-associated yeasts. (**B**) PLS-DA analysis illustrating separation of yeast isolates based on isolation origin. Number (n) of representative yeast genomes are indicated in the lower corners. PERMANOVA was used for statistical analysis (p < 0.001 for all genera). (**C**) Total genome length across isolation source and yeast genera. Statistical analysis conducted using Mann–Whitney U and Bonferroni correction, with significant differences indicated by different letters (p < 0.05). *Aureobasidium* isolates from flower and *Metschnikowia* isolates from human origin were unavailable and were therefore only represented by four bars. (**D**) Glycoside hydrolase gene distribution across environments. (**E–F**) Orthogroup analysis identifying genes or gene clusters that contribute to the observed separation in PLS-DA plot for (**E**) *Aureobasidium* and (**F**) *Rhodotorula*. (**G**) Network analysis of 290 annotated genes associated with leaf adaptation in *Aureobasidium*. Single cluster analysis was performed using Cytoscape software with the GlueGO plugin. Functional annotation based on GO terms and KEGG pathways. Only the label of the most significant term per functional group shown (Benjamini-Hochberg, p < 0.05 and Kappa score level ≥ 0.4). Edges represent functional associations between genes, indicating that connected genes share similar biological processes or participate in related metabolic pathways

We next sought to identify genomic signatures of phyllosphere yeasts for *Aureobasidium*, *Metschnikowia*, *Candida*, and *Rhodotorula* (from the wheat phyllosphere and NCBI derived genomes) by clustering all genes across genera into orthogroups and subsetting the data per genus (Fig. 4A). The primary factor driving differences across isolates was taxonomy, however, supervised classification based on orthogroup count, using PLS-DA, revealed statistically significant clustering by isolation source across all four genera (Fig. 4B, Supplementary Fig. 7A; PERMANOVA p < 0.001). Pairwise comparisons showed significant separation between leaf and human isolates for *Aureobasidium*, *Candida*, and *Rhodotorula*, while differences between leaf and soil were observed for *Aureobasidium* and *Candida*. Furthermore, we also found a significant separation between *Metschnikowia* isolates originating from leaf and water (Supplementary Fig. 7A, B; Pairwise PERMANOVA p < 0.05). The genome size of leaf-associated *Aureobasidium* isolates was significantly larger than that of human- and soil-associated *Aureobasidium* isolates, whereas no difference in genome size between the isolates of the other genera from different origin was observed (Fig. 4C; Mann Whitney U and Bonferroni correction, p = 0.02). We also observed a significant increase in the number of glycoside hydrolases in leaf-associated yeasts (Fig. 4D; Mann Whitney U with Bonferroni correction, p < 0.05). These differences are partly explained by species-specific differences in *Aureobasidium*, since most leaf-associated yeasts belong to *A. pullulans*, while yeasts from other environments were annotated as *A. melanogenum* and *A. zeae* (Supplementary Fig. 8).

Thereafter, we performed a targeted pangenome analysis by pooling the orthogroups of all genera from the same environment. A total of 853,022 genes (99.4%) were distributed across 18,563 orthogroups, while 5,003 genes remained unassigned. Comparative genome analysis was performed based on presence and absence of orthogroups compared to the ‘soft core’ (*i.e.* 95%, based on 1,185 orthogroups). We determined the number of unique orthogroups for each isolation source, and all possible isolation source combinations (Supplementary Fig. 9). Due to these strict requirements, 15,816 orthogroups could not be placed in any category, while a core of 1,185 was observed, followed by 383 shared orthogroups for all sources except water. The phyllosphere samples, *i.e.* leaf and flower, shared 96 orthogroups. Interestingly, we also identified unique orthogroups for the phyllosphere environment, with 107 unique orthogroups for leaf-associated yeasts, and 106 unique orthogroups for flower-associated yeasts. In addition, a total of 33 orthogroups were shared between leaf and soil samples.

Next, we examined whether functional categories were enriched or depleted in the different origins using the COG database. We investigated the shared orthogroups between leaf-flower (96 orthogroups, 705 COG hits), leaf-soil (33 orthogroups, 691 COG hits), and leaf-soil-flower (85 orthogroups, 212 COG hits), as these environments are ecologically more similar relative to human- and water-associated habitats (Supplementary Fig. 9; Fisher’s test and Benjamini–Hochberg correction, odds ratio < -50% and > 50%, p < 0.05). We focused on functional categories with over- or underrepresentation based on at least 200 genes to obtain enough statistical power. Functional (COG) categories such as carbohydrate metabolism and transport, and secondary metabolism (biosynthesis, transport, and catabolism) were overrepresented in the unique orthogroups of leaf-associated yeasts (Supplementary Fig. 10). Unique orthogroups of flower-associated yeasts were enriched in carbohydrate metabolism and transport, replication, recombination and repair, and post-translational modification, protein turnover and chaperones, and depleted in signal transduction mechanisms. The overrepresentation of carbohydrate metabolism and transport was also apparent in orthogroups shared between leaf and soil samples. Additionally, secondary metabolites were overrepresented in the orthogroups shared between leaf-soil, and leaf-flower-soil (Supplementary Fig. 10).

Given the overrepresentation of secondary metabolites biosynthesis, transport, and catabolism in leaf-associated yeasts, we used the fungiSMASH database to identify putative plant-adaptive BGCs. Large variations in total number of BGCs were observed across genera, regardless of the origin (Supplementary Fig. 11). Due to the low number of BGCs detected by fungiSMASH (Supplementary Fig. 5 and 11), we also employed KEGG pathway completeness analysis to investigate if the unique orthogroups found in leaf, flower, soil, human, and water samples, were associated with similar or unique biosynthetic pathways. In leaf-associated yeasts, three metabolic pathways were more complete compared to other isolation origins, which included GABA biosynthesis (in eukaryotes), ammonia dissimilation, and pectin degradation (Supplementary Fig. 12B). Further investigation revealed that *Aureobasidium* was the primary driver of these differences, potentially due to its larger genome size and higher representation among leaf-associated yeasts (Supplementary Fig. 12A, C). To account for this, we conducted a separate analysis employing each genus and isolation source independently. Mean gene counts per orthogroup were used to compare the differences between leaf-associated and all other environments, for each genus separately (Fig. 4E, 4F). A total of 1,110 orthogroups were significantly enriched or depleted in leaf-associated *Aureobasidium*, representing 1577 hypothetical genes, of which 290 were annotated (Fig. 4E, G). A network analysis using Cytoscape, based on KEGG and GO annotation, revealed an enrichment of genes involved in organic substance transport, cellular nitrogen compound catabolism, and metabolic processes related to nucleic acid, single-organism metabolism, organic substances, and cellular nitrogen compounds (Fig. 4G). Contrarily, for *Rhodotorula* (Fig. 4F) and *Candida* no significantly different orthogroups were observed, while only two orthogroups were significantly different present in *Metschnikowia* (Wilcoxon and Benjamini–Hochberg correction; p < 0.05).

**Fig. 5:**
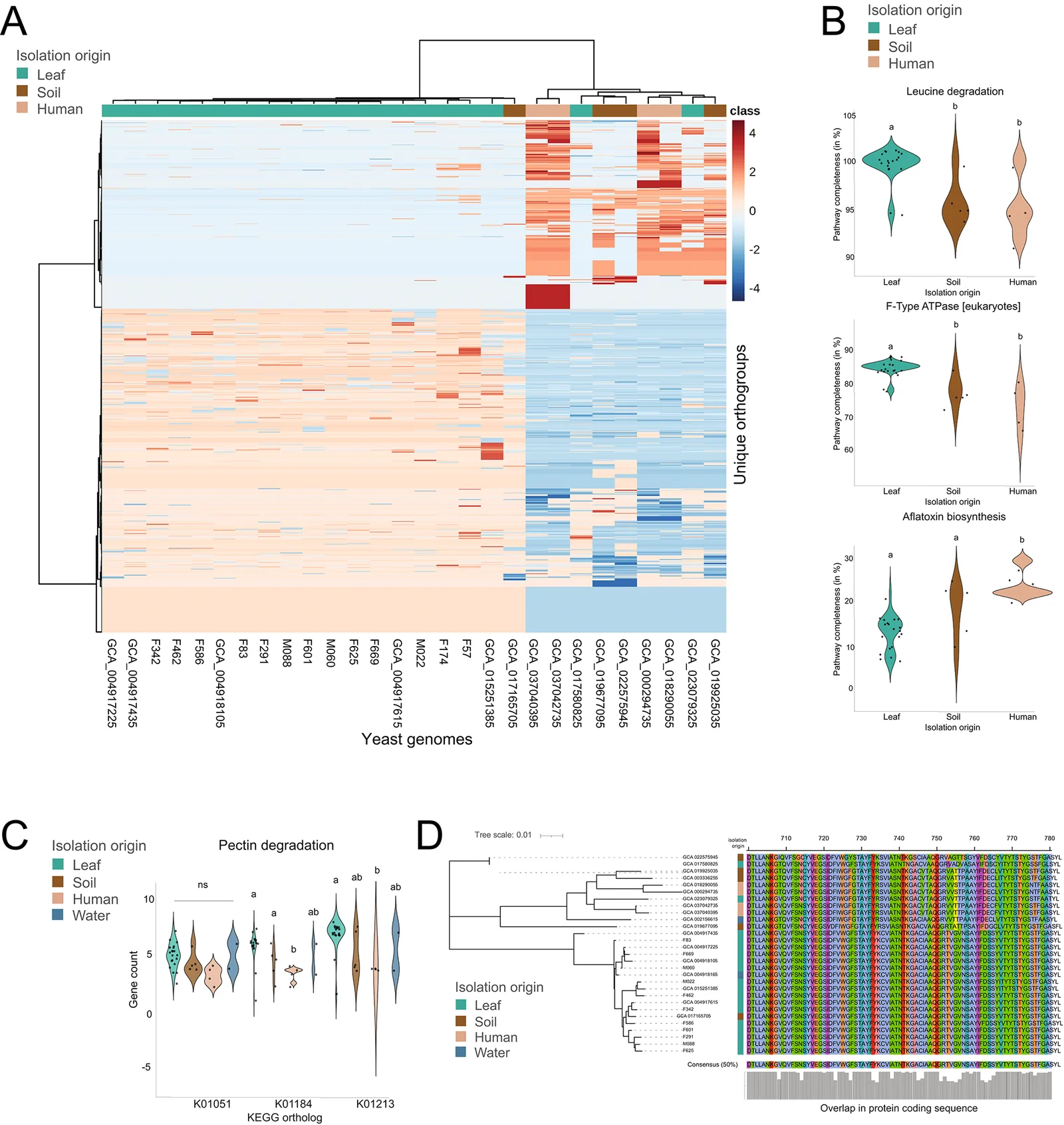
Pathway completeness analysis of *Aureobasidium* genomes. (**A**) Heatmap showing differences in pathway completeness between leaf-associated isolates and isolates from soil- and human origins. Fifteen pathways were significantly enriched in one of the isolation sources (Mann Whitney U, Bonferroni, p < 0.05). (**B**) Enrichment of genes involved in leucine degradation and F-type ATPase for leaf-associated isolates and in aflatoxin biosynthesis for human-associated isolates identified by pathway completeness analysis. (**C**) Enrichment in gene count per genome for enzymes involved in pectin degradation (K01051, K01184, K01213). Significant differences indicated by * or different letters (p < 0.05) based on Mann Whitney U and Bonferroni correction. (**D**) Pectin methylesterase genes present in all *Aureobasidium* isolates, with variations in the protein-coding sequence at multiple locations. Isolation origins are color-coded and shown on the left side of each panel. Due to the limited number of water-associated *Aureobasidium* isolates (n = 2), it was not included in the heatmap (A) and violin plots (B, C). Differences in protein sequences are highlighted by amino acids in distinct colors. The bar graph indicates consensus between protein sequences

We observed the strongest separation among leaf-associated yeasts in PCA analysis for *Aureobasidium*, with 1,110 orthogroups significantly different across isolation sources (Fig. 4B, 5A). These differences were driven by 15 pathways, including leucine degradation (a potential energy source) and F-type ATPase (energy production; eukaryotes), enriched in leaf-associated yeasts, and aflatoxin biosynthesis (a toxic secondary metabolite) enriched in human-associated yeasts (Fig. 5B). Additionally, the pectin degradation pathway, which was previously observed across all genera was also highlighted, with pectinase genes found among the enriched genes in leaf-associated *Aureobasidium*. We further investigated the pectin degradation pathway, where a higher gene count was observed between leaf-, soil-, and human-associated *Aureobasidium* (Mann–Whitney U with Bonferroni correction, p < 0.05). Examining the protein coding sequence revealed differences in protein domains between leaf-associated and other environmental isolates (Fig. 5C). Notable substitutions, such as alanine to threonine, serine to cysteine, or tyrosine to leucine were identified (Fig. 5D).

Next, we explored whether the phylogenetic clustering observed for the pectin methylesterase gene is driven by taxonomic relationships (Supplementary Fig. 13). By comparing the phylogenetic tree derived from the 758 BUSCO genes with the phylogeny based on the pectin methylesterase gene, we identified two distinct clusters. As observed before species-specificity could explain the differences in *Aureobasidium*. Specifically, the first cluster predominantly contained leaf-associated yeasts assigned to *Aureobasidium pullulans*, while the second cluster was mainly composed of yeasts from other isolation origins, including *A. melanogenum* along with several other species. This separation suggests that species-level selection pressures could influence the ability of *A. pullulans* to colonize leaf environments.

To further investigate whether the genetic differences distinguishing leaf-associated yeasts could be attributed to species- or cluster-level factors rather than environment factors alone, we conducted an additional analysis at the genus and cluster levels. The genome dataset was unavoidably shaped by source bias and uneven representation across isolation sources—particularly within specific genus-cluster combinations—which limited the statistical power for formal inference. To address this, we employed a targeted approach. Orthogroup counts were grouped by source type (Leaf vs. Others), and mean values were calculated separately for each genus and cluster combination (Supplementary Fig. 14). We specifically examined four orthogroups (OG0000270, OG0000019, OG0000056, OG0000455) that were enriched in leaf-associated genomes (Supplementary Fig. 15, Supplementary Table 8). Their distribution across genus-cluster combinations was visualized using boxplots with jittered points to highlight variability in gene counts between Leaf and Others groups. Orthogroups 000455 and 000270 were of specific interest, separating leaf-associated yeasts from other sources for *Metschnikowia*, and *Candida*, respectively. Functional analysis based on KEGG pathways of these groups indicated their involvement in various molecular processes, where orthogroup OG000455 includes genes predicted as dihydrolipoyl dehydrogenase, which plays a vital role in energy metabolism, while orthogroup OG000270 includes genes predicted as histone binding proteins, which are involved in DNA organization.

## Discussion

Yeasts thrive in diverse environments, including plants, soil, water, and extreme habitats such as Antarctica or desserts [7]. This remarkable adaptative ability to prosper in various environments is most likely due to their broad substrate utilization spectrum, tolerance to different abiotic environmental conditions and competitiveness in biotic interactions. Our previous meta-survey of yeast genomes from the wheat phyllosphere revealed the largely unknown functional potential of yeasts [22]. Here, we broadened the catalogue of phyllosphere-associated yeasts with nearly 100 high-quality genome assemblies, enabling unprecedented analysis of the intra- and inter-diversity of 14 different genera. By adding 96 new genomes, we have doubled the number of *Aureobasidium, Candida,* and *Metschnikowia* genomes from phyllosphere environments, providing the foundation for studying yeast ecology and adaptive traits in the dynamic phyllosphere habitat. Despite significantly expanding genomic resources for phyllosphere-associated yeasts, many core yeast and fungal members (*e.g. Ustilago*, *Malassezia*, *Itersonilia*) remain underrepresented in genome databases. Here, our newly acquired genomes were included in a comparative analysis with 106 publicly available high-quality reference yeast genomes to identify phyllosphere-adaptive traits.

The wide taxonomic diversity of yeasts in the wheat phyllosphere was reflected by the large putative functional properties of the various genera. Significant phyllosphere specialization was observed for smaller genomes (*e.g. Candida* and *Metschnikowia*) with an overrepresentation of eight and four out of the 23 COG functional categories, respectively, including secondary metabolism, as well as nucleotide, amino acid, and carbohydrate metabolism. These smaller genomes may have undergone functional streamlining and specialization, focusing their genetic repertoire on specific metabolic processes that are essential for survival in their ecological niche. This specialization might also reflect a trade-off, where smaller genomes optimize for efficiency and specific functionality at the expense of broader metabolic versatility [43]. The overrepresentation of categories such as secondary metabolism as well as nucleotide, amino acid, and carbohydrate metabolism may suggest an evolutionary prioritization of pathways that provide competitive advantages in the phyllosphere.

The discrepancy between the overrepresentation of secondary metabolism in *Candida* and *Metschnikowia* and the very low number of BGCs observed with fungiSMASH highlights the untapped biotechnological potential of these phyllosphere yeasts as well as the limited resolution of current bioinformatic tools for the detection and annotation of these BGCs. FungiSMASH works primarily for well-documented yeasts, such as *S. cerevisiae*, or the biotechnologically important *Aureobasidium*, which might suggest the production of novel secondary metabolites, or different metabolic pathways in *Candida* and *Metschnikowia* which are not yet correctly identified by FungiSMASH [23]. On the other hand, EggNOG provides holistic functional annotations, which may lead to inflated or incorrect predictions, especially in specialized areas of metabolism. Additionally, the smaller genomes of *Candida* and *Metschnikowia* might harbor smaller cryptic gene clusters, or gene clusters integrated into other biosynthetic pathways, making them harder to detect with current algorithms [24]. Moreover, the overrepresentation of secondary metabolism based on COG categories could reflect the production of small metabolites that enhance metabolic flexibility or environmental adaptation, rather than classic bioactive secondary metabolism. This highlights the need for alternative computational approaches to uncover the full biosynthetic potential of phyllosphere and other environmental yeast genera.

To identify genomic signatures of leaf-associated yeasts, we analyzed four genera with at least 15 available genomes, resulting in a curated dataset of 128 high-quality genomes from diverse environments. Using machine learning based on random forest and support vector machine models, to identify genomic features predictive of isolation origin, we showed that classification accuracy varied by yeast genus. The drop of classification performance from reference to LOGO models highlights the overfitting of the reference model and the putative lack of a universal genomic signature for leaf association. The model performed best when *Rhodotorula* was excluded, while exclusion of *Aureobasidium* led to a sharp decline, suggesting distinct genomic adaptations in *Aureobasidium*. *Metschnikowia* and *Candida* showed weaker predictive value, and no universal leaf-associated signature emerged. These findings highlight the need for genus-specific approaches rather than cross-genus models, particularly given the limitation of representative genomes. The availability of more genomes will allow detailed genome-wide association mapping of both coding and non-coding regions can better reveal the interplay between environmental adaptation and genome evolution [25]. Additionally, higher yeast genome numbers will allow computational methods to reduce the dominant (and confounding) phylogenetic signals, enabling the study of subtle differences, such as leaf-associated genome selection, using cross-genus universal models. While in our study we explored leaf-associated genome selection as an individual trait, previous research has shown that microbe–microbe interactions also play an important role in shaping microbial phenotypes [26, 27]. Therefore, future modeling efforts should investigate how community-level traits influence the genomic adaptation of individual members. In addition, although all genome assemblies were annotated using the same computational pipeline to minimize biases in gene detection, differences in sequencing platforms, assembly quality, and the inclusion or absence of supporting data (e.g., transcriptomics) can lead to variation in the final predicted proteomes. This inherent limitation should be taken into consideration when interpreting comparative analyses.

We found that taxonomic identity explained most of the differences between yeast orthogroup distribution. To a lesser extent, isolation origin also explained part of the differential separation between the yeast isolates. This highlights the presence of genes, gene clusters, or pathways that may enhance survival or increase colonization in different ecological niches. For example, *Aureobasidium* is widely distributed across diverse environments, including soil, water, plants and indoor areas. Their widespread distribution suggests that they have versatile ecological roles in biodegradation and plant–microbe interactions. Functional (COG) categories, such as carbohydrate metabolism and transport, and secondary metabolites biosynthesis, transport, and catabolism, were overrepresented in leaf-associated yeasts. Overrepresentation of carbohydrate metabolism and transport has also been observed for plant-associated bacteria [12, 15], which was hypothesized to be driven by the more chemically complex organic carbons present on leaves. However, no mechanistic studies have been performed yet to validate these computational findings. Our study is first to report an overrepresentation of genes involved in secondary metabolite biosynthesis, transport, and catabolism in leaf-associated yeasts. In addition to being exposed to other microorganisms in the phyllosphere, these yeasts also maintain close interactions with their plant hosts. As such, some of the genes and gene clusters belonging to these COG or CAZyme categories may play a role in plant-yeast interactions, either facilitating a beneficial relationship or serving as a defense mechanism against toxic plant compounds. Experimental validation, encompassing site-directed mutagenesis and heterologous expression of target genes followed by elaborate plant-yeast interaction assays, is needed to explore the exact functional implications of these phyllosphere adaptive traits. For some of the yeast genera identified in this study, we started to employ a range of mutagenesis strategies for the identified gene clusters. However, the transformation of these environmental yeast genera/strains was not yet feasible, potentially due to their intrinsic resistance to available genetic manipulation [28]. Our previous and present studies support the idea that co-occurring yeast isolates from the same host may adopt distinct metabolic [29] and evolutionary strategies to allow their coexistence in the phyllosphere niche; this differentiation likely contributes to the limited detection of universal genomic signatures associated with phyllosphere adaptation across genera.

Genus-specific differences were evident, as *Aureobasidium* contributed disproportionately to leaf-specific signatures, with enrichment in genes associated with organic substance transport, nitrogen metabolism, and pectin degradation. Phylogenetic clustering of the pectin methylesterase gene revealed a clear separation of leaf-associated *A. pullulans* from other *Aureobasidium* species, suggesting species-level selection pressures that may drive adaptation to the phyllosphere. These findings suggest that phyllosphere adaptation in yeasts is largely genus- or even species-specific, rather than governed by universal genomic signatures. The presence of distinct phylogenetic clusters based on the pectin methylesterase gene may reflect adaptation to the specific ecological niches of leaves, allowing *A. pullulans* to thrive in this environment, while other species are more suited to different habitats. However, whether this specialization results from a direct selection pressure or other factors remains to be investigated. This underscores the need for lineage-specific genomic and functional analyses to elucidate the evolutionary trajectories and ecological roles of yeasts in plant-associated environments. Our comparative genome analysis offers important insights into the adaptations of yeasts to the phyllosphere environment. However, the dataset exhibits a degree of bias, with an overrepresentation of Ascomycota genomes and limited coverage of key members of the core microbiota (e.g., *Dioszegia*, *Filobasidium*, *Papiliotrema*, *Sporobolomyces*, and *Vishniacozyma*). These taxa are well represented in our culture collection but currently lack whole-genome sequences from other plant, human, and soil sources. Out of the four genera included in our comparative analysis, only *Aureobasidium* was consistently detected as a core member of the wheat phyllosphere microbiome, highlighting the transient and context-dependent nature of the phyllosphere, and the difficulty of identifying consistent selection pressures across genera. Although *Candida* was absent from our amplicon survey, multiple *Candida* strains were recovered through isolation, suggesting primer bias. Including *Candida* in our comparative genomics provides a valuable reference for lineage-specific adaptations and underscores the need to expand genome resources for less dominant but potentially important phyllosphere yeasts. Despite these limitations, our study represents an important step toward understanding yeast adaptation to the phyllosphere, and increasing genome availability will further clarify the genetic traits underlying survival and specialization in this environment.

In conclusion, our work greatly expanded the availability of phyllosphere-associated yeast genomes, laying the ground for unprecedented comparative genome analyses. The observed inter- and intra-specific diversity underscores the largely untapped functional potential of these phyllosphere yeasts despite the limitations of currently available databases and computational tools. By integrating the newly sequenced genomes with other high-quality genomes from public repositories, we identified key traits and pathways that may facilitate colonization in the harsh phyllosphere environment. Notably, the protein-coding difference in pectin methyl-esterases across leaf-associated *Aureobasidium* presents an exciting starting point for future experimental validation. The availability of nearly 100 phyllosphere yeast isolates, along with their genomic data, will drive future functional studies of the crop phyllosphere microbiome. Furthermore, the genomic insights obtained in this study are significant to provide a better understanding of the diversity and activity of yeasts in the phyllosphere, which could pave the way for novel (bio)technological applications.

## Methods

### Sample collection and DNA extractions

Flag leaves from field-grown (Taastrup, Denmark) winter wheat (*Triticum aestivum*) cultivars Elixer, Rembrandt, Kvium, Heerup and Sheriff were collected for yeast and pathogenic fungi community profiling. Individual flag leaves collected in June 2020 (Elixer), were transferred to 50 mL tubes with 0.9% NaCl and 0.05% Tween20 and vortexed for 2 min, sonicated for 2 min at 45 kHz, and vortexed again for 2 min. For DNA extraction, the resulting pellets were resuspended in 978 µL PBS, transferred to lysis tubes, and mixed with 122 µL of C1 solution from the DNeasy Powersoil Kit, followed by manufacturer’s instructions (Qiagen, Hilden, Germany). Individual flag leaves collected in June 2021 (Rembrandt, Kvium, Heerup and Sheriff) were freeze-dried and crushed before subjecting 15 mg (± 2 mg) to DNA extraction using the Mag-Bind Plant DNA DS Kit (Omega Bio-Tek, Norcross, Georgia). DNA concentrations were assessed using a Qubit 2.0 fluorometer (Invitrogen, Eugene, Oregon, US).

Extracted DNA was used for yeast and pathogenic fungi community profiling via metabarcoding of the ITS1 region. Library preparation was carried out using a two-step PCR approach. The first PCR amplified the ITS1 region with primers ITS1F-KYO2 and ITS86R, each containing overhangs for subsequent indexing [30, 31]. Reactions were conducted on an Eppendorf Mastercycler Nexus X2 Thermal Cycler (Eppendorf, Hamburg, Germany), and contained 12.5 µL Platinum Hot Start PCR 2 × Master Mix (Invitrogen, Eugene, Oregon, US), 0.5 µL Bovine Serum Albumin (BSA; 20 mg/mL) (Thermo Fisher Scientific, Massachusetts, US), 0.2 µM of each primer, 3 µL template DNA (< 0.05–2.5 ng DNA), and DNase free water to a final volume of 25 µL. Thermocycling conditions were 94 °C for 2 min, followed by 35 cycles of 94 °C for 30 s, 55 °C for 30 s and 72 °C for 40 s with a final extension at 72 °C for 10 min. PCR products were verified on a 1% agarose gel, purified using magnetic AMPure XP beads in a ratio of beads:sample of 0.6:1 (Beckman Coulter, Brea, CA, US), and eluted in 20 µL DNAse-free water [32]. Indexing was performed in a second PCR using 5 µL of the first AMPure XP bead-cleaned PCR product as template. Illumina indexing primers were used which carry barcodes and indexes for sequencing along with homology to the 5' overhangs of the primers used in the first PCR. Forward primer i5/P5: 5′-AATGATACGGCGACCACCGAGATCTACAC-[i5]-TCGTCGGCAGCGTC-3′, and reverse primer i7/P7 5′-CAAGCAGAAGACGGCATACGAGAT-[i7]-GTCTCGTGGGCTCGG-3′. The second PCR followed the same reaction setup and cycling conditions, but 0.8 µM of each barcoded index primer was used, no BSA was added, and the number of cycles were limited to 15. Final products were purified using magnetic AMPure XP beads in a ratio of beads:sample of 1:1 (Beckman Coulter, Brea, CA, US), and eluted in 15 µL DNAse-free water. Concentrations of the final amplicons were quantified on a Qubit 2.0 fluorometer (Invitrogen, Eugene, Oregon, US). Libraries were pooled in equimolar concentrations and sequenced on an Illumina MiSeq platform with 2 × 300 bp paired-end reads using the MiSeq Reagent Kit V3 following manufacturer’s instructions (Illumina Inc., San Diego, California, US).

### Yeast and pathogenic fungi community profiling

Demultiplexed reads from each collection year were processed separately using the DADA2 pipeline (v1.26.0) in R (v4.2.3) to infer amplicon sequence variants (ASVs) [33]. The standard DADA2 workflow was followed, including quality filtering, error model learning, denoising, paired-end read merging, and chimera removal. During quality filtering, reads with quality scores below 30 were trimmed using the filterAndTrim function, and reads with > 2 expected errors in forward or > 3 in reverse reads (as set by the maxEE parameter) were discarded. ASVs were inferred using pooled sample inference with default dereplication and denoising settings. Taxonomy was assigned at the species level using a naive Bayesian classifier against the SILVA database (release 138.1), and taxonomy-assigned data were imported into a phyloseq object [34]. Data from individual years were then merged across two years using the mergeSequenceTables function (dada2 package v1.48) [33]. ASVs classified as mitochondria, protoplasts, singletons or undefined at the genus level were removed from the abundance table. 

Alpha diversity metrics (Observed and Shannon indices) were calculated using the estimate_richness function from the phyloseq package [35]. For multivariate community analyses, pairwise Bray–Curtis dissimilarity was computed, and principal coordinate analysis (PCoA) was performed using the dist_calc and ord_calc functions from the microViz package (v0.12.4) [36]. PERMANOVA was conducted using the adonis2 function from the vegan package (v2.6.8) [37], and environmental variables were fitted to the ordination axes with the envfit function, with significance determined by permutation testing. The top five environmental variables for three clusters were selected using k-means clustering from the stats package (v4.4.2) and visualized as vectors with the geom_segment function in ggplot2 (v3.5.1). To identify genera associated with collection year, differential abundance analysis was performed using ANCOM-BC2 (v2.6.0) [38].

### Isolation of yeasts from wheat flag leaves

Yeasts were isolated from the flag leaves of wheat (*Triticum aestivum*) cultivars Elixer, Rembrandt, Kvium, Heerup, and Sheriff as described previously [29]. Briefly, leaves were washed or ground in 0.9% NaCl + 0.05% Tween20. Serial dilutions were plated on a range of general and selective media (MEA, PDA, SDA, YEPD, and 869 media supplemented with flag leaf extract), with and without the addition of 10% lactic acid. Media were supplemented with adding 50 µl/ml chloramphenicol and 50 µl/ml tetracycline to prevent bacterial growth. After incubation at 25ºC for 5–7 days, colonies were purified and cultivated on YEPD plates or broth, and preserved in 15% glycerol at -70ºC. Additionally, an enrichment protocol was included to increase taxonomic diversity. Ground flag leaf material was inoculated in Potato Dextrose Broth (PDB) and Minimal media supplemented with 1% methanol (common carbon source for microorganisms inhabiting the leaves) to promote yeast growth. Bacterial growth was prevented by adding 50 µl/ml chloramphenicol and 50 µl/ml tetracycline. Cultures were incubated at 10 °C and 20 °C at 180 rpm for 7–10 days before plating. Thereafter, tenfold serial dilutions were prepared, and 100 µl was plated on Potato Dextrose Agar and on Minimal media [39] supplemented with 1% methanol and 1.8% agar. Plates were incubated for 5 to 7 days at the corresponding temperature (enrichment period). Yeast colonies were picked and streaked on fresh plates (at least twice) to obtain pure cultures. Isolate stocks were prepared from agar plates and stored in 15% glycerol (v/v), all samples were stored at -70 °C for long-term storage.

### gDNA extraction and genome sequencing

DNA was extracted with the Wizard Genomic DNA purification kit (Promega). Yeasts, previously isolated from the wheat flag leaf, were grown for 2–7 days on Potato Dextrose Agar (PDA, pH 7) until sufficient biomass was available. Cells were scraped from the plates and ground until fine powder by mortar and pestle by the addition of liquid nitrogen. Around 250 µl of powder was added to a 2.0 mL Eppendorf tube with 900 µL of Nuclei Lysis Solution, and tubes were inverted 20 times. Three hundred µL of Protein Precipitation Solution was added and vortexed vigorously at high speed for 20 s and samples were left on ice for 5 min. Samples were centrifuged for 10 min at 16.000 rpm and the supernatant was transferred to a clean 1.5 mL Eppendorf tube containing 900 µL of isopropanol at RT. Samples were inverted 20 times and centrifuged for 10 min at 16.000 rpm. The supernatant was removed, 900 µL of 70% ethanol at RT was added and the tubes were inverted ten times. The samples were centrifuged for 5 min at 16.000 rpm and all ethanol was removed by pipetting and air-drying the sample for 15 min. DNA was rehydrated in 50 µL of DNA Rehydration solution containing 1.5 µL of RNAse solution. The sample was vortexed for 1 s and incubated at 37 °C for 15 min. The DNA was further rehydrated overnight at 4 °C and stored at -70 °C. Concentrations and integrity were measured by Nanodrop (Thermo Fisher ND-One) and Fragment Analyzer (Agilent 5200).

Samples were sequenced by a combination of Nanopore and Illumina sequencing yielding 44 genomes (Supplementary Fig. 3). Quality control of Illumina raw and Nanopore sequencing reads was performed using Fastp (0.23.2) [40]. The other 52 yeast genomes were sequenced using PacBio sequencing. Genomic DNA was subjected to quality control using SMRTbell™ cleanup beads, damaged and end repairs were conducted prior to SMRTbell library construction. Libraries were sequenced on the PacBio Sequel System using P6-C4 chemistry.

### Genome assembly and phylogenetic analysis

Post-processing quality assessment was conducted using MultiQC v1.12 [41], which utilized the combination of long reads Nanopore sequencing and short reads from Illumina sequencing. Long read sequencing data were basecalled using Guppy (Oxford Nanopore Technologies Ltd.) and filtered with’fitlong’. The initial long read assembly was conducted using Flye (version 2.9.3-b1797) [200], followed by consensus sequence generation with Medaka (Oxford Nanopore Technologies Ltd.) to refine the Flye assembly. Subsequently, short reads were integrated using Pilon [201] for assembly correction. The quality of genome assemblies was assessed using the BUSCO v5.6.1 (Benchmarking Universal Single-Copy Orthologs) [42] assessment tool, using the ODb10 Fungi dataset. Additional BUSCO analyses performed using the Ascomycota and Basidiomycota lineage datasets were consistent with the results obtained with the general Fungi dataset (data not shown). During the assembly quality assessment using the BUSCO program (version BUSCO 5.6.1), assemblies F297 and F355 were identified to have a high percentage of duplicate genes, prompting further investigation. The underlying causes for the poor initial assemblies were investigated; assembly F297 was compromised by low sequencing depth, and F355 was suspected to be a polyploid. The assemblies were further analyzed using GenomeScope (v2.0) and Smudgeplot (v0.2.5) [29], though no definitive conclusions could be drawn. Consequently, both F297 and F355 were reassembled using SPAdes genome assembler (v3.15.5) [30]. The assembly of the sequencing output from PacBio was done using Flye, after converting PacBio bam formats to fastq using bamtools [43]. Quast was used to assess the quality of the assemblies providing assembly quality metrics such as N50 and contig counts [44]. The completeness of the assemblies was further evaluated using BUSCO, checking against conserved orthologs to gauge assembly completeness.

Phylogenomic analyses was performed using BUSCO_phylogenomics [45]. The database used was the fungi_odb10, a total of 758 single copy marker genes compared between the isolates using the mafft Multiple Sequence Alignment (MSA). The phylogenomic trees were reconstructed using IQ-TREE (multicore version 2.3.3) using default parameters [46], and the iTol v7 software was used for visualization [47].

### Genome annotation

Genome assemblies were annotated using Funannotate version 1.8.17 [48]. Initially, repetitive sequences in the genome were masked using funannotate mask. Following the masking, paired-end transcriptomic data, available for 15 isolates from a different experiment, were aligned to the genome using hisat2-build and hisat2 for indexing and alignment, respectively [49]. The resulting SAM files were converted to BAM format, sorted, and indexed using samtools [50]. For the isolates with transcriptomic data available, de novo transcriptome assembly was performed using Trinity [51] to enhance the accuracy of subsequent annotation. Gene prediction models were trained with RNA-seq data via funannotate train and integrated along with Trinity assemblies to predict genes using funannotate predict, with the ‘Fungi’ database [52–54]. If transcriptomic data was not available, the pipeline performed gene prediction directly with funannotate predict. EggNOG-mapper [55, 56] provided functional annotations.

### Orthologous group identification

Orthofinder [57] was employed for orthogroup inference to further understand the evolutionary relationships and functional similarities among the predicted genes. OrthoFinder utilizes gene predictions generated by the funannotate program, conducting all-vs-all sequence similarity searches followed by clustering to infer orthogroups, thereby providing insights into gene families and their evolutionary histories. By clustering genes into orthogroups, we gained a holistic view of gene content and diversity across species, facilitating comprehensive pangenomic analyses that reveal both core and accessory genetic elements. Unique sets of orthogroups were identified per genera, along with the corresponding genes from each species composing the different orthogroups, which were annotated based on Cluster of Orthologous Groups (COGs) and functional annotations [58].

### Functional over- and under-representation analysis

To identify overrepresented and underrepresented COGs among the different subsets of isolates, we conducted a functional enrichment analysis. COG functional annotation for all orthogroups (unique and combinations) was compared to those of the core orthogroups, present in 95% of the species. Fisher’s exact test was used to determine significance of COG enrichment compared to the core genes. We compared the count of genes in the core group to the count in each specific group by constructing a contingency table for each comparison and computing the odds ratio and p-value using Fisher Exact statistical test. To account for multiple comparisons, we applied the Benjamini–Hochberg correction to the p-values, controlling the false discovery rate. This adjustment ensured that the reported p-values reflect the adjusted significance levels. Additionally, we calculated the log odds ratio (LOR) for each comparison to facilitate the interpretation of the enrichment direction and magnitude.

### Pathway completeness analysis

To assess pathway completeness, we first identified orthogroups present in at least 95% of the source isolates. From these orthogroups, we extracted a list of KEGG Orthology (KO) identifiers based on their annotations. This KO list was then used as input for the KEGG Pathways Completeness Tool (https://github.com/EBI-Metagenomics/kegg-pathways-completeness-tool) to evaluate the completeness of metabolic pathways across the analyzed isolates.

### NCBI genome database, origin, and dereplication

We manually curated a list of available genomes from the NCBI database, gathering all genome assemblies from the 14 yeast genera. Available metadata was used to determine the isolation source, which were categorized into phyllosphere, flower, water, soil, human, abiotic, and other environments. Genomes with known isolation sources were retained, while low-quality genomes (BUSCO completeness < 75%) were removed. For phylogenomic analyses, we used the same procedure as mentioned above. To remove redundancy, genomes with ≥ 99% sequence similarity were dereplicated. Only genera represented by at least 20 genomes were included to ensure sufficient statistical power. The final filtered set of publicly available genomes was re-annotated using the genome annotation pipeline described in the Methods, which integrates Funannotate and EggNOG-mapper without incorporating transcriptomic evidence.

To assess the environmental distribution of the 14 yeast genera, we analyzed data from the GlobalFungi database [21], consolidating environments into eight categories: dead organic material (*e.g.* dead wood and litter), dust, lichens and mosses, soil and roots (*e.g.* root, rhizosphere soil, soil and topsoil), sediment, shoot and leaves, and water. Categories with fewer than 20 occurrences, including coral, glacial ice debris, and fungal sporocarp, were excluded from the analysis.

### Machine learning models

Genomic data and orthogroup gene counts were obtained from the OrthoFinder pipeline (v2.5.5) output and preprocessed for downstream analysis [57]. Isolates were classified into two categories via a binary system (*e.g.* leaf-associated were assigned 1 and non-leaf-associated assigned 0) based on predefined taxonomic assignments. Supervised classification models were trained using a Random Forest (RF) classifier with 100 estimators and a Support Vector Machine (SVM) classifier with a linear kernel, both implemented in scikit-learn (v1.5.2) [59]. We implemented Random Forest (RF) (RandomForestClassifier, sklearn.ensemble) and Support Vector Machine (SVM) (SVC, sklearn.svm) classifiers to infer the genomic signatures of leaf-associated yeasts. Model performance was assessed using repeated stratified k-fold cross-validation (sklearn.model_selection RepeatedStratifiedKFold, n_splits = 5, n_repeats = 10) to ensure robust generalization. The RF classifier was trained using 100 decision trees. The SVM classifier was implemented using a linear kernel with scikit-learn’s SVC. Classification accuracy was estimated through cross-validation, with mean accuracy and standard deviation computed across iterations. To determine the most informative orthogroups for classification, SHapley Additive exPlanations (SHAP) (v0.47.0) (shap.explainer function) was used. The mean absolute SHAP values were calculated for each orthogroup, ranking features based on their contribution to classification. The top 100 most informative orthogroups for each class (leaf-associated and non-leaf-associated) were extracted.

The same dataset as described above was used for the leave one genus out (LOGO) model. Besides the binary classification, strains were grouped into four genera: *Aureobasidium*, *Candida*, *Metschnikowia*, and *Rhodotorula* to implement Leave-One-Genus-Out Cross-Validation (LOGO-CV). For each iteration, genomes belonging to a single genus were set aside as a test set, while the remaining genera formed the training set. The models were trained on the training set and tested on the held-out genus.

### Statistical analysis

Statistical testing was performed in RStudio 2023.06.1 + 524. Principal component analysis (PCA) and supervised Partial Least Squares Discriminant Analysis (PLS-DA) were performed using prcomp() and plsda(), in combination with ggplot() prompts. PERMANOVA was performed using manova() and adonis2(). Shapiro–Wilk test was used to test for normality via shapiro.test(). When data were normally distributed, ANOVA with Tukey HSD were used via aov() and TukeyHSD(), while for abnormally distributed, the non-parametric Wilcoxon test was used via wilcox.test() and Bonferroni for correction of multiple testing via p.adjust, method = ‘bonferroni’.

## Supplementary Information

Supplementary Material 1: Supplementary Figure 1. Statistical analysis of yeast andpathogenic fungi community profiling . A) Shannon index and observed ASV counts in 2020(orange) and 2021 (blue). Statistical significance is based on Wilcoxon test and differencesare indicated by an asterisk (p = 0.0002). B) Bar plot of relative abundance of yeast genera(logFC) in 2021 compared to 2020 (enriched; in red, depleted; in blue).

Supplementary Material 2: Supplementary Figure 2. Relative abundance in the yeast and pathogenic fungi community of isolated yeast genera. The relative abundance of 10 yeast genera, present in the isolate collection, observed in the ITS amplicon sequence data in 2020 and 2021.

Supplementary Material 3: Supplementary Figure 3. Phylogenetic tree of 96 wheat phyllosphere yeasts. Maximum likelihood phylogeny based on 758 BUSCO single-copy genes. The first circle shows the taxonomic group, where color-coding follows the same order as presented in the legend, followed by harvest year, and sequencing technique. Quality control was visualized using BUSCO analysis in a stacked bar plot, and number of contigs (blue). Genome architecture was shown with genome size (in Mb; orange), GC content (in %; purple), and N50 (green). Color stripes highlight low to higher values, ranging from light to dark color codes.

Supplementary Material 4: Supplementary Figure 4. Functional category abundances based on Clusters of Orthologous Genes (COGs). A) Differences in COG categories between yeast genera. The number of unique (blue bars) orthogroups or combinations (green bars) between genera are shown in the Upset plot on the left. B) Heatmaps indicate the level of over- and underrepresentation (at least < -50% or > 50%) of significant COGs. Pink and red-colored cells indicate overrepresentation, while purple-colored cells represent under representation compared to the soft core of all pangenomes (95%, based on 1966 orthogroups).

Supplementary Material 5: Supplementary Figure 5. Overview of Biosynthetic Gene Clusters (BGCs) identified in the phyllosphere yeast genomes. A) BGC distribution based on fungiSMASH tool. The isolates are ordered based on phylogeny, and the number of each BGC hit, subdivided by type, are color coded. Bar plot on the right side represents the total number of BGCs per category, while the bar plot on top represents the number of BGCs per isolate. B) Compound hits provided by fungiSMASH, blocks are colored based on BGC type and identity similarity, ranging from 12 – 100% (light to dark colored). Hybrid indicates a secondary metabolite with at least two BGC type hits.

Supplementary Material 6: Supplementary Figure 6. Diversity in carbohydrate degradation enzymes across phyllosphere yeast genomes. The Carbohydrate-Active Enzymes (CAZyme) database was employed to profile the diversity of polysaccharide degrading enzymes. The total number of CAZymes (from 0 – 30 genes per category) present per isolate, subdivided into auxiliary activities, carbohydrate-binding molecules, carbohydrate27esterases, glycosyltransferases, and polysaccharide lyases. The stacked bar plot indicates number of CAZymes (color-coded based on type) normalized by genome size.

Supplementary Material 7: Supplementary Figure 7. Phylogeny and environmental distribution of yeasts. A) PLS-DA analysis based on orthogroup count per yeast genera, representing the largest components. Statistical analysis was conducted using B) Pairwise PERMANOVA comparing isolation sources across each genus. Statistical significance indicated in green (p < 0.05). NaN = less than 3 representatives available for statistical analysis. Color coding is the same for each PCA plot.

Supplementary Material 8: Supplementary Figure 8. Phylogenetic analysis, based on orthogroup gene count, and PCA separation. Yeast genera were subdivided into clusters, which were used for PCA analysis separation. Origin of isolate based on color-coding (leaf; green, flower; magenta, aqua; blue, soil; brown, and human in beige.

Supplementary Material 9: Supplementary Figure 9. Unique and shared orthogroups between genomes of yeasts from different isolation sources. A total of 18.563 orthogroups were identified for 128 yeast isolates. Strict clustering based on > 95% occurrence resulted in a core of 1185 orthogroups. The top bar plot indicates the number of orthogroups per combination, while the bar plot on the left indicates the total number of orthogroups per isolation source.

Supplementary Material 10: Supplementary Figure 10. Functional category abundance. Differences in gene categories (COGs) between leaf-, flower-, soil-, human-, and water-associated yeasts. Number of genes with functional annotation (left) for each unique isolation origin, and for the combination leaf-flower, leaf-soil, and leaf-flower-soil. Pink-colored cells indicate significant overrepresentation (> 50%) and purple-colored cells indicate significant underrepresentation (< -50%) compared to the core (95%, based on 2076 orthogroups). Lower bar graph indicates number of genes per functional category. Cells represented by only a few genes are more likely to be less accurate. Bold categories refer to those over- or under represented in leaf-associated yeasts, which are represented by at least 200 genes.

Supplementary Material 11: Supplementary Figure 11. Overview of Biosynthetic Gene Clusters (BGCs) identified in *Aureobasidium *and *Rhodotorula*. BGC distribution based on fungiSMASH tool in *Aureobasidium* (A) and *Rhodotorula *(C). Statistical differences are based on Wilcoxon with Bonferroni correction, differences are indicated by different letters for *Aureobasidium *(B) and *Rhodotorula *(D). Bar plot indicates the total number of BGC hits per strain, bars are colored based on isolation origin. E) Compound hits provided by fungiSMASH, blocks are colored based on BGC type and identity similarity, ranging from 12 – 100% (light to dark colored). Bar plot indicates the total number of identified BGCs per strain, bars are colored based on isolation origin.

Supplementary Material 12: Supplementary Figure 12. Taxonomic analysis of drivers associated with the separation of leaf- and other-associated yeasts. A) PCA plot based on orthogroup gene count, dots are color coded based on28isolation origin. B) Mean pathway completeness of leaf-associated yeast across all genera vs mean pathway completeness of yeasts from all other sources (in %). C) Violin plots showing mean pathway completeness of overrepresented pathways in leaf-associated yeasts, as indicated in panel B. Dots are color-coded based on genera, and violin plots separated based on isolation origin.

Supplementary Material 13: Supplementary Figure 13. Phylogenetic analyses of yeasts based on 758 BUSCO genes and a pectin methylesterase gene. Phylogenetic analysis was performed in MEGA, and Muscle alignment. Isolate and accession numbers are color-coded based on isolation origin (leaf; green, flower; magenta, aqua; water, soil; brown, and human in beige.

Supplementary Material 14: Supplementary Figure 14. Orthogroups enriched in leaf-associated yeasts. Counts of orthogroups in leaf- and others-associated yeasts, with strains grouped according to the clustering observed in Supplementary Figure 8. Orthogroups significantly enriched in leaf-associated yeasts are highlighted in green, orthogroups labeled in black were selected for additional analysis in Supplementary Figure 15.

Supplementary Material 15: Supplementary Figure 15. Orthogroup analysis of leaf-associated yeasts. Orthogroup counts of leaf and others-associated yeasts, subdivided in clusters, of significantly different orthogroups observed in Supplementary Figure 14. Bars are color-coded by isolation origin; green for leaf-associated yeasts and gray for yeasts from other environments.

Supplementary Material 16. Supplementary Table 1. DADA2 output for amplicon sequences from field-grown wheat flag leaves from 2020 and 2021.

Supplementary Material 17. Supplementary Table 2. Relative abundance of yeast ASVs detected in the wheat flag leaf (after removal of low abundant ASVs).

Supplementary Material 18. Supplementary Table 3. Taxonomy and accession numbers of type strains and wheat phyllosphere strains (this study) used for the phylogenetic analysis.

Supplementary Material 19. Supplementary Table 4. Overview of yeast isolates from the wheat phyllosphere used for whole genome sequencing (columns A, B). Field year and wheat cultivars (columns C, D), sequencing platforms (columns F-G) and genome statistics (columns H-P).

Supplementary Material 20. Supplementary Table 5. Yeast genomes and accession numbers from NCBI.

Supplementary Material 21. Supplementary Table 6. Training and testing set models used for the Leave-One-Group-Out (LOGO) cross-validation approach.

Supplementary Material 22. Supplementary Table 7. Accuracies, operating characteristic (ROC) curves for reference and LOGO models (RF: Random Forest, SVM: Support Vector Machine).

Supplementary Material 23. Supplementary Table 8. Functions of the genes belonging to orthogroups enriched in leaf-associated genomes.

## Data Availability

The datasets generated and analyzed during the current study are available in the ENA repository, project accession PRJEB88777 (ERP171839).
