# Supplementary figures and images for: Genomic insights into adaptative traits of phyllosphere yeasts
Source: Environ Microbiome. 2026 Jan 3;21:21. doi: 10.1186/s40793-025-00839-7 (PMC12866564; doi:10.1186/s40793-025-00839-7)

A

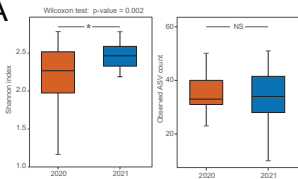

B

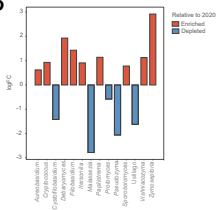

Supplement: Supplementary file 1 — Supplementary Material 1: Supplementary Figure 1. Statistical analysis of yeast andpathogenic fungi community profiling . A) Shannon index and observed ASV counts in 2020(orange) and 2021 (blue). Statistical significance is based on Wilcoxon test and differencesare indicated by an asterisk (p = 0.0002). B) Bar plot of relative abundance of yeast genera(logFC) in 2021 compared to 2020 (enriched; in red, depleted; in blue). [file 40793_2025_839_MOESM1_ESM.pdf]

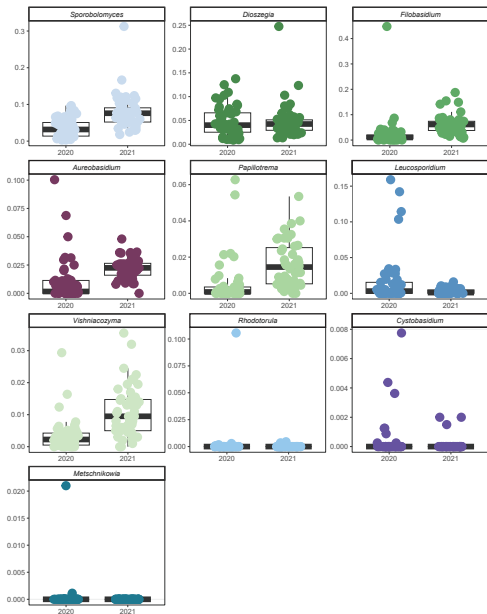

Supplement: Supplementary file 2 — Supplementary Material 2: Supplementary Figure 2. Relative abundance in the yeast and pathogenic fungi community of isolated yeast genera. The relative abundance of 10 yeast genera, present in the isolate collection, observed in the ITS amplicon sequence data in 2020 and 2021. [file 40793_2025_839_MOESM2_ESM.pdf]

A

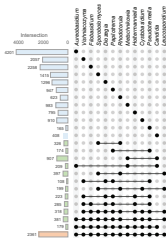

B

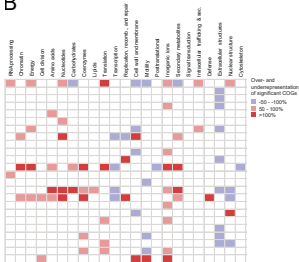

Supplement: Supplementary file 4 — Supplementary Material 4: Supplementary Figure 4. Functional category abundances based on Clusters of Orthologous Genes (COGs). A) Differences in COG categories between yeast genera. The number of unique (blue bars) orthogroups or combinations (green bars) between genera are shown in the Upset plot on the left. B) Heatmaps indicate the level of over- and underrepresentation (at least < -50% or > 50%) of significant COGs. Pink and red-colored cells indicate overrepresentation, while purple-colored cells represent under representation compared to the soft core of all pangenomes (95%, based on 1966 orthogroups). [file 40793_2025_839_MOESM4_ESM.pdf]

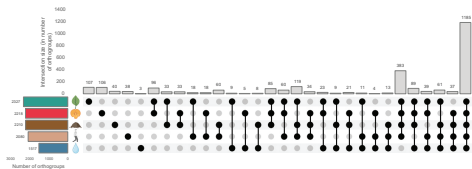

Supplement: Supplementary file 9 — Supplementary Material 9: Supplementary Figure 9. Unique and shared orthogroups between genomes of yeasts from different isolation sources. A total of 18.563 orthogroups were identified for 128 yeast isolates. Strict clustering based on > 95% occurrence resulted in a core of 1185 orthogroups. The top bar plot indicates the number of orthogroups per combination, while the bar plot on the left indicates the total number of orthogroups per isolation source. [file 40793_2025_839_MOESM9_ESM.pdf]

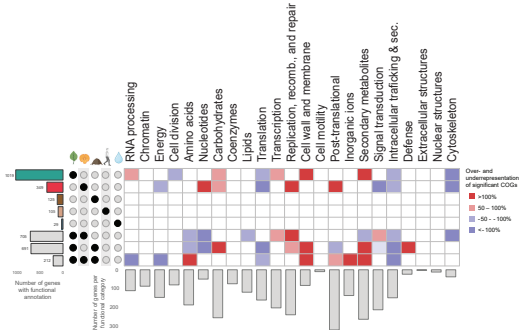

Supplement: Supplementary file 10 — Supplementary Material 10: Supplementary Figure 10. Functional category abundance. Differences in gene categories (COGs) between leaf-, flower-, soil-, human-, and water-associated yeasts. Number of genes with functional annotation (left) for each unique isolation origin, and for the combination leaf-flower, leaf-soil, and leaf-flower-soil. Pink-colored cells indicate significant overrepresentation (> 50%) and purple-colored cells indicate significant underrepresentation (< -50%) compared to the core (95%, based on 2076 orthogroups). Lower bar graph indicates number of genes per functional category. Cells represented by only a few genes are more likely to be less accurate. Bold categories refer to those over- or under represented in leaf-associated yeasts, which are represented by at least 200 genes. [file 40793_2025_839_MOESM10_ESM.pdf]

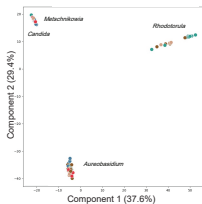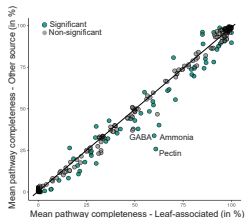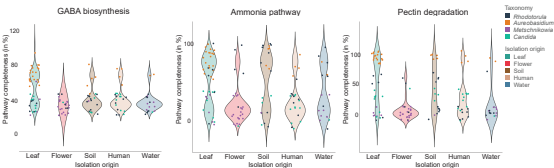

Supplement: Supplementary file 12 — Supplementary Material 12: Supplementary Figure 12. Taxonomic analysis of drivers associated with the separation of leaf- and other-associated yeasts. A) PCA plot based on orthogroup gene count, dots are color coded based on28isolation origin. B) Mean pathway completeness of leaf-associated yeast across all genera vs mean pathway completeness of yeasts from all other sources (in %). C) Violin plots showing mean pathway completeness of overrepresented pathways in leaf-associated yeasts, as indicated in panel B. Dots are color-coded based on genera, and violin plots separated based on isolation origin. [file 40793_2025_839_MOESM12_ESM.pdf]

# Phylogeny based on BUSCO

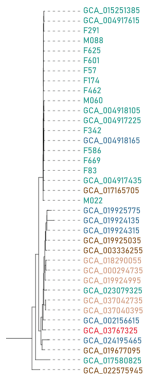

# Phylogeny based on pectin methylesterase

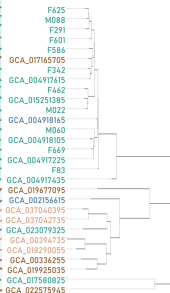

Supplement: Supplementary file 13 — Supplementary Material 13: Supplementary Figure 13. Phylogenetic analyses of yeasts based on 758 BUSCO genes and a pectin methylesterase gene. Phylogenetic analysis was performed in MEGA, and Muscle alignment. Isolate and accession numbers are color-coded based on isolation origin (leaf; green, flower; magenta, aqua; water, soil; brown, and human in beige. [file 40793_2025_839_MOESM13_ESM.pdf]

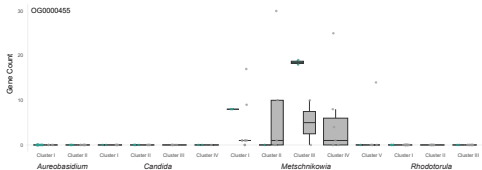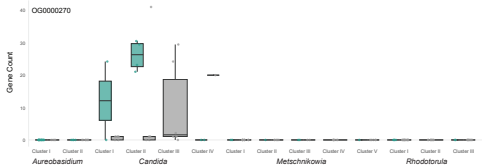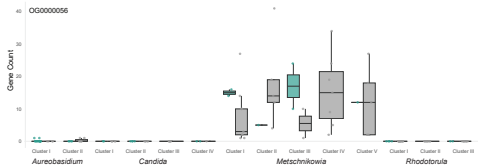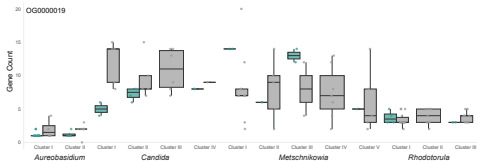

Isolation origin Leaf Other sources

Supplement: Supplementary file 15 — Supplementary Material 15: Supplementary Figure 15. Orthogroup analysis of leaf-associated yeasts. Orthogroup counts of leaf and others-associated yeasts, subdivided in clusters, of significantly different orthogroups observed in Supplementary Figure 14. Bars are color-coded by isolation origin; green for leaf-associated yeasts and gray for yeasts from other environments. [file 40793_2025_839_MOESM15_ESM.pdf]
