# Supplementary material for: Genomic insights into adaptative traits of phyllosphere yeasts
Source: Environ Microbiome. 2026 Jan 3;21:21. doi: 10.1186/s40793-025-00839-7 (PMC12866564; doi:10.1186/s40793-025-00839-7)
Supplement: Supplementary file 3 — Supplementary Material 3: Supplementary Figure 3. Phylogenetic tree of 96 wheat phyllosphere yeasts. Maximum likelihood phylogeny based on 758 BUSCO single-copy genes. The first circle shows the taxonomic group, where color-coding follows the same order as presented in the legend, followed by harvest year, and sequencing technique. Quality control was visualized using BUSCO analysis in a stacked bar plot, and number of contigs (blue). Genome architecture was shown with genome size (in Mb; orange), GC content (in %; purple), and N50 (green). Color stripes highlight low to higher values, ranging from light to dark color codes. [file 40793_2025_839_MOESM3_ESM.pdf]

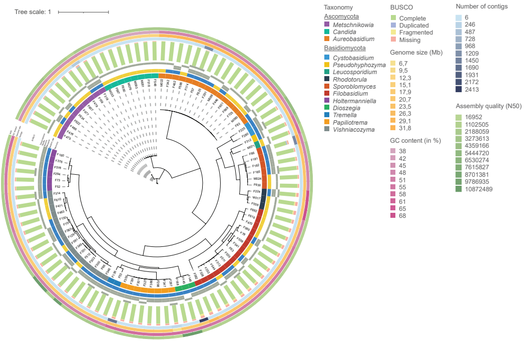

Tree scale: 1
Taxonomy
Ascomycota
Metschnikowia
Candida
Aureobasidium
Basidiomycota
Cystobasidium
Pseudohyphozyma
Leucosporidium
Rhodotorula
Sporoblomyces
Filobasidium
Holtermanniella
Dioszegia
Tremella
Papiliotrema
Vishniacozyma
BUSCO
Complete
Duplicated
Fragmented
Missing
Genome size (Mb)
6,7
9,5
12,3
15,1
17,9
20,7
23,5
26,3
29,1
31,8
GC content (in %)
38
42
45
48
51
55
58
61
65
68
Number of contigs
6
246
487
728
968
1209
1450
1690
1931
2172
2413
Assembly quality (N50)
16952
1102505
2188059
3273613
4359166
5444720
6530274
7615827
8701381
9786935
10872489
