# Supplementary material for: Genomic insights into adaptative traits of phyllosphere yeasts
Source: Environ Microbiome. 2026 Jan 3;21:21. doi: 10.1186/s40793-025-00839-7 (PMC12866564; doi:10.1186/s40793-025-00839-7)
Supplement: Supplementary file 6 — Supplementary Material 6: Supplementary Figure 6. Diversity in carbohydrate degradation enzymes across phyllosphere yeast genomes. The Carbohydrate-Active Enzymes (CAZyme) database was employed to profile the diversity of polysaccharide degrading enzymes. The total number of CAZymes (from 0 – 30 genes per category) present per isolate, subdivided into auxiliary activities, carbohydrate-binding molecules, carbohydrate27esterases, glycosyltransferases, and polysaccharide lyases. The stacked bar plot indicates number of CAZymes (color-coded based on type) normalized by genome size. [file 40793_2025_839_MOESM6_ESM.pdf]

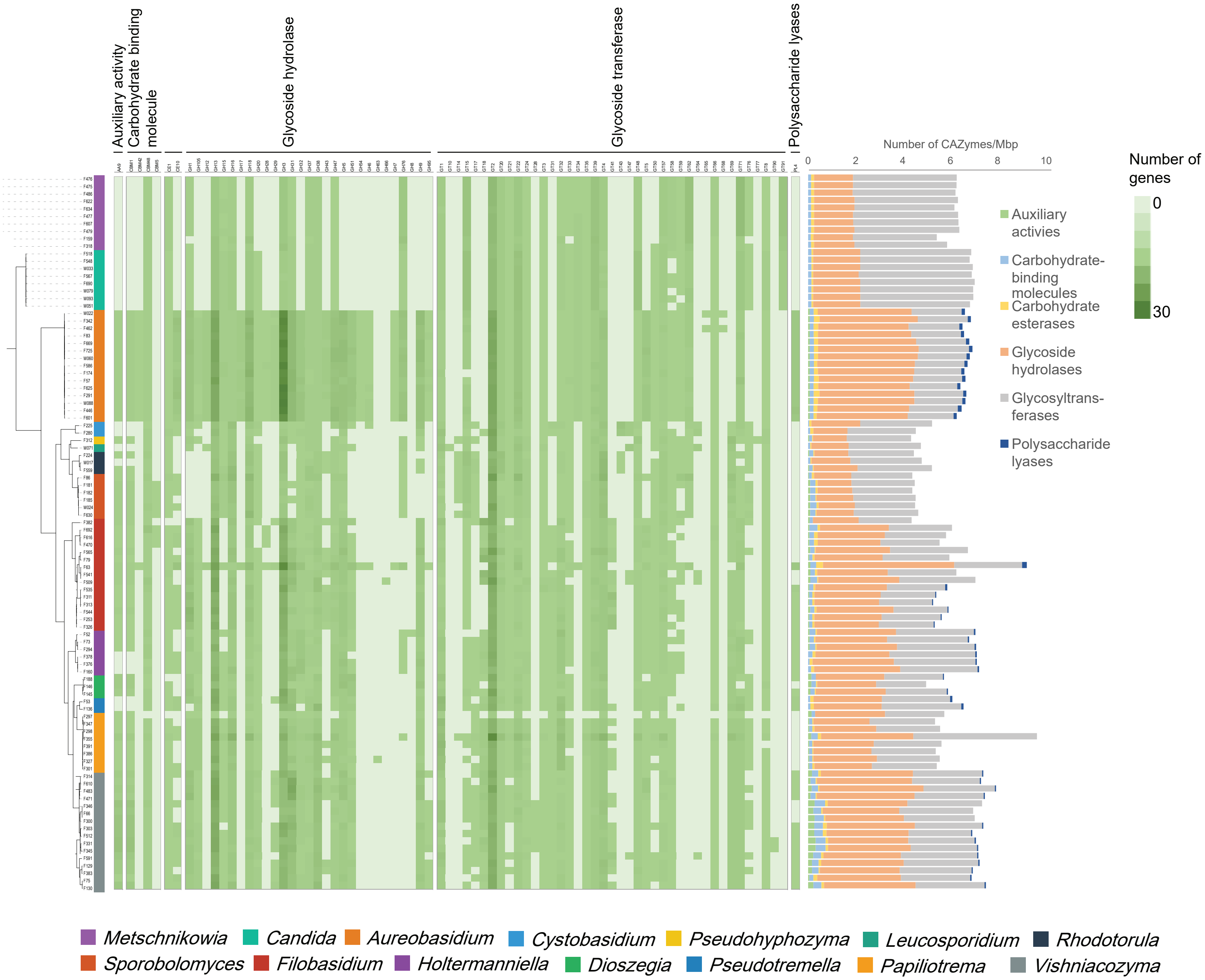
Auxiliary activity
Carbohydrate binding molecule
Glycoside hydrolase
Glycoside transferase
Polysaccharide lyases
Number of CAZymes/Mbp
0
2
4
6
8
10
Number of genes
0
30
Auxiliary activies
Carbohydrate-binding molecules
Carbohydrate esterases
Glycoside hydrolases
Glycosyltrans-ferases
Polysaccharide lyases
Metschnikowia
Candida
Aureobasidium
Cystobasidium
Pseudohyphozyma
Leucosporidium
Rhodotorula
Sporobolomyces
Filobasidium
Holtermanniella
Dioszegia
Pseudotremella
Papiliotrema
Vishniacozyma
