# Supplementary material for: Genomic insights into adaptative traits of phyllosphere yeasts
Source: Environ Microbiome. 2026 Jan 3;21:21. doi: 10.1186/s40793-025-00839-7 (PMC12866564; doi:10.1186/s40793-025-00839-7)
Supplement: Supplementary file 7 — Supplementary Material 7: Supplementary Figure 7. Phylogeny and environmental distribution of yeasts. A) PLS-DA analysis based on orthogroup count per yeast genera, representing the largest components. Statistical analysis was conducted using B) Pairwise PERMANOVA comparing isolation sources across each genus. Statistical significance indicated in green (p < 0.05). NaN = less than 3 representatives available for statistical analysis. Color coding is the same for each PCA plot. [file 40793_2025_839_MOESM7_ESM.pdf]

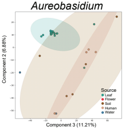

	Leaf	Flower	Soil	Human	Water	n
Leaf	NaN					6
Flower	0.026	NaN				20
Soil	NaN	NaN	NaN			1
Human	NaN	NaN	NaN	NaN		-
Water	0.002	0.004	NaN	NaN	NaN	7

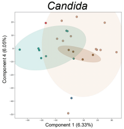

	Leaf	Flower	Soil	Human	Water	n
Leaf	NaN					8
Flower	NaN	NaN				1
Soil	0.034	NaN	NaN			4
Human	0.036	NaN	0.237	NaN		11
Water	NaN	NaN	NaN	NaN	NaN	1

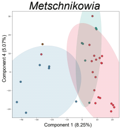

	Leaf	Flower	Soil	Human	Water	n
Leaf	NaN					6
Flower	0.026	NaN				20
Soil	NaN	NaN	NaN			1
Human	NaN	NaN	NaN	NaN		-
Water	0.002	0.004	NaN	NaN	NaN	7

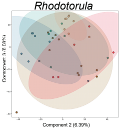

	Leaf	Flower	Soil	Human	Water	n
Leaf	NaN					6
Flower	0.352	NaN				5
Soil	0.199	0.155	NaN			11
Human	0.009	0.003	0.060	NaN		8
Water	0.522	0.068	0.164	0.058	NaN	8
