# Supplementary material for: Genomic insights into adaptative traits of phyllosphere yeasts
Source: Environ Microbiome. 2026 Jan 3;21:21. doi: 10.1186/s40793-025-00839-7 (PMC12866564; doi:10.1186/s40793-025-00839-7)
Supplement: Supplementary file 8 — Supplementary Material 8: Supplementary Figure 8. Phylogenetic analysis, based on orthogroup gene count, and PCA separation. Yeast genera were subdivided into clusters, which were used for PCA analysis separation. Origin of isolate based on color-coding (leaf; green, flower; magenta, aqua; blue, soil; brown, and human in beige. [file 40793_2025_839_MOESM8_ESM.pdf]

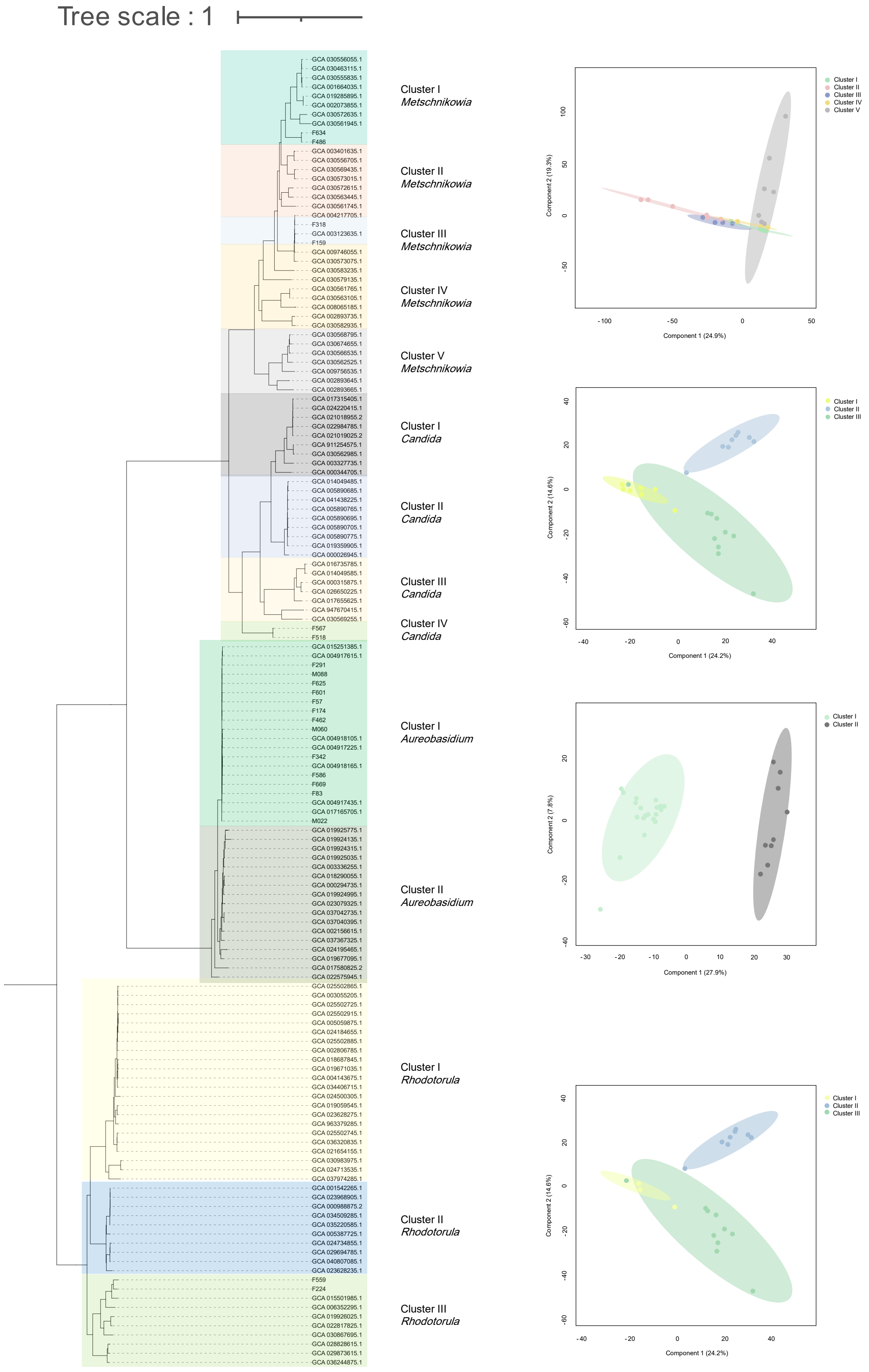

Tree scale : 1
Cluster I Metschnikowia
Cluster II Metschnikowia
Cluster III Metschnikowia
Cluster IV Metschnikowia
Cluster V Metschnikowia
Cluster I Candida
Cluster II Candida
Cluster III Candida
Cluster IV Candida
Cluster I Aureobasidium
Cluster II Aureobasidium
Cluster I Rhodotorula
Cluster II Rhodotorula
Cluster III Rhodotorula
Component 1 (24.9%)
Component 2 (19.3%)
Component 1 (24.2%)
Component 2 (14.6%)
Component 1 (27.9%)
Component 2 (7.8%)
Cluster I
Cluster II
Cluster III
Cluster IV
Cluster V
