# Supplementary material for: Genomic insights into adaptative traits of phyllosphere yeasts
Source: Environ Microbiome. 2026 Jan 3;21:21. doi: 10.1186/s40793-025-00839-7 (PMC12866564; doi:10.1186/s40793-025-00839-7)
Supplement: Supplementary file 14 — Supplementary Material 14: Supplementary Figure 14. Orthogroups enriched in leaf-associated yeasts. Counts of orthogroups in leaf- and others-associated yeasts, with strains grouped according to the clustering observed in Supplementary Figure 8. Orthogroups significantly enriched in leaf-associated yeasts are highlighted in green, orthogroups labeled in black were selected for additional analysis in Supplementary Figure 15. [file 40793_2025_839_MOESM14_ESM.pdf]

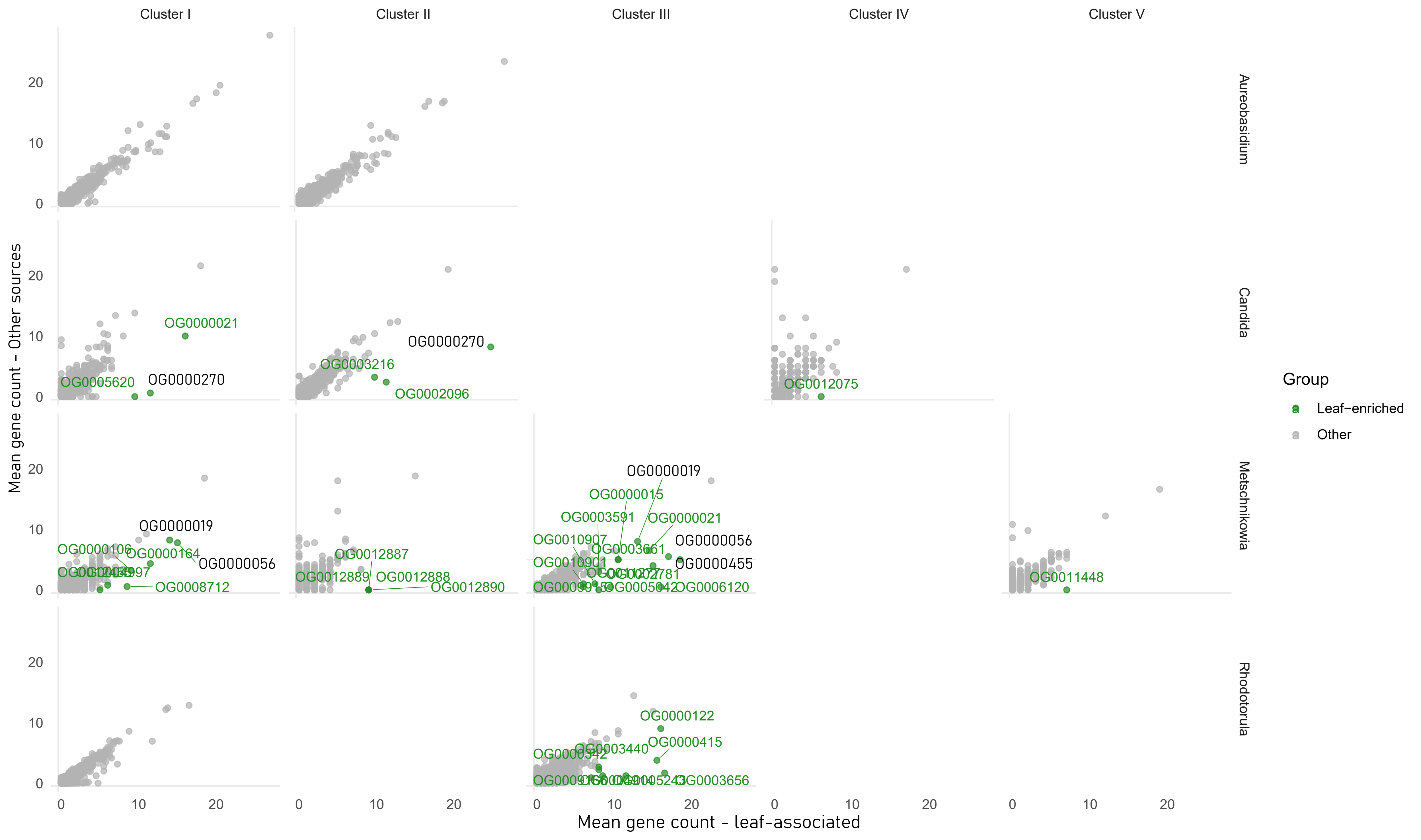

Cluster I
Cluster II
Cluster III
Cluster IV
Cluster V
Aureobasidium
Candida
Metschnikowia
Rhodotorula
Mean gene count - Other sources
Mean gene count - leaf-associated
Group
Leaf−enriched
Other
OG0000021
OG0005620
OG0000270
OG0003216
OG0002096
OG0012075
OG0000019
OG0000164
OG0000056
OG0008712
OG0012887
OG0012889
OG0012888
OG0012890
OG0000015
OG0003591
OG0010907
OG0003661
OG0010901
OG0000455
OG0006120
OG0011448
OG0000122
OG0000415
OG0003440
OG0003656
