# Supplementary material for: Genomic insights into adaptative traits of phyllosphere yeasts
Source: Environ Microbiome. 2026 Jan 3;21:21. doi: 10.1186/s40793-025-00839-7 (PMC12866564; doi:10.1186/s40793-025-00839-7)
Supplement: Supplementary file 5 — Supplementary Material 5: Supplementary Figure 5. Overview of Biosynthetic Gene Clusters (BGCs) identified in the phyllosphere yeast genomes. A) BGC distribution based on fungiSMASH tool. The isolates are ordered based on phylogeny, and the number of each BGC hit, subdivided by type, are color coded. Bar plot on the right side represents the total number of BGCs per category, while the bar plot on top represents the number of BGCs per isolate. B) Compound hits provided by fungiSMASH, blocks are colored based on BGC type and identity similarity, ranging from 12 – 100% (light to dark colored). Hybrid indicates a secondary metabolite with at least two BGC type hits. [file 40793_2025_839_MOESM5_ESM.pdf]

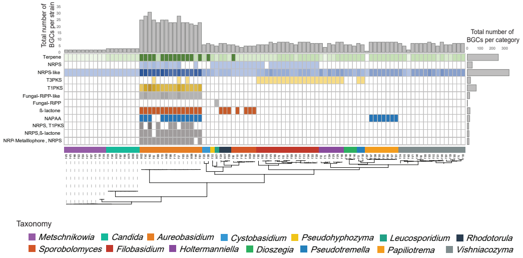

Total number of BGCs per strain
Total number of BGCs per category
Terpene
NRPS
NRPS-like
T3PKS
T1PKS
Fungal-RiPP-like
Fungal-RiPP
ß-lactone
NAPAA
NRPS, T1PKS
NRPS,ß-lactone
NRP-Metalllophore , NRPS
Taxonomy
Metschnikowia
Candida
Aureobasidium
Cystobasidium
Pseudohyphozyma
Leucosporidium
Rhodotorula
Sporobolomyces
Filobasidium
Holtermanniella
Dioszegia
Pseudotremella
Papiliotrema
Vishniacozyma

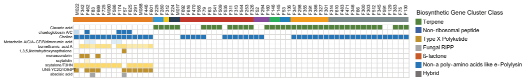

Clavaric acid
chaetoglobosin A/C
Choline
Metachelin A/C/A-CE/B/dimerumic acid
burnettramic acid A
1,3,6,8-tetrahydroxynapthalene
monascorubrin
scytalidin
scytalone/T3HN
UNII-YC2Q1O94PT
abscisic acid
Biosynthetic Gene Cluster Class
Terpene
Non-ribosomal peptide
Type X Polyketide
Fungal RiPP
ß-lactone
Non-a poly-amino acids like e-Polylysin
Hybrid
