# Supplementary material for: Genomic insights into adaptative traits of phyllosphere yeasts
Source: Environ Microbiome. 2026 Jan 3;21:21. doi: 10.1186/s40793-025-00839-7 (PMC12866564; doi:10.1186/s40793-025-00839-7)
Supplement: Supplementary file 11 — Supplementary Material 11: Supplementary Figure 11. Overview of Biosynthetic Gene Clusters (BGCs) identified in Aureobasidium and Rhodotorula. BGC distribution based on fungiSMASH tool in Aureobasidium (A) and Rhodotorula (C). Statistical differences are based on Wilcoxon with Bonferroni correction, differences are indicated by different letters for Aureobasidium (B) and Rhodotorula (D). Bar plot indicates the total number of BGC hits per strain, bars are colored based on isolation origin. E) Compound hits provided by fungiSMASH, blocks are colored based on BGC type and identity similarity, ranging from 12 – 100% (light to dark colored). Bar plot indicates the total number of identified BGCs per strain, bars are colored based on isolation origin. [file 40793_2025_839_MOESM11_ESM.pdf]

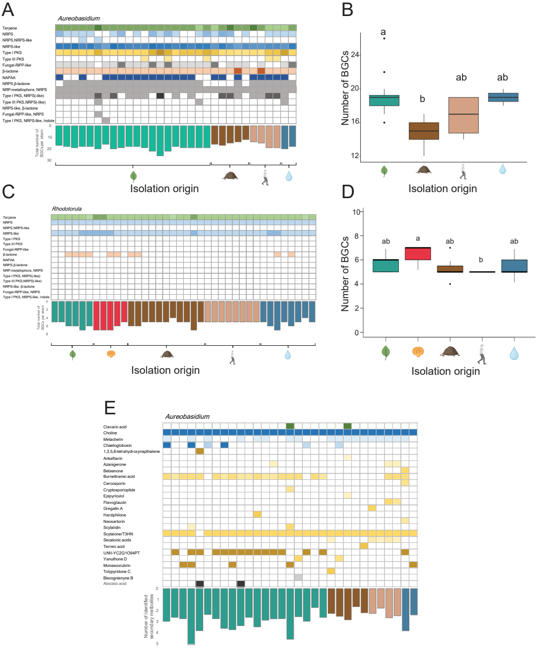
A
Aureobasidium
Terpene
NRPS
NRPS,NRPS-like
NRPS-like
Type I PKS
Type III PKS
Fungal-RiPP-like
β-lactone
NAPAA
NRPS,β-lactone
NRP-metallophore, NRPS
Type I PKS, NRPS(-like)
Type III PKS,NRPS(-like)
NRPS-like, β-lactone
Fungal-RiPP-like, NRPS
Type I PKS, NRPS-like, Indole
Total number of BGCs per strain
0
10
20
30
Isolation origin
B
Number of BGCs
12
16
20
24
a
b
ab
ab
Isolation origin
C
Rhodotorula
Terpene
NRPS
NRPS,NRPS-like
NRPS-like
Type I PKS
Type III PKS
Fungal-RiPP-like
β-lactone
NAPAA
NRPS,β-lactone
NRP-metallophore, NRPS
Type I PKS, NRPS(-like)
Type III PKS,NRPS(-like)
NRPS-like, β-lactone
Fungal-RiPP-like, NRPS
Type I PKS, NRPS-like, Indole
Total number of BGCs per strain
0
2
4
6
8
Isolation origin
D
Number of BGCs
0
2
4
6
8
10
ab
a
ab
b
ab
Isolation origin
E
Aureobasidium
Clavaric acid
Choline
Metachelin
Chaetoglobosin
1,3,5,8-tetrahydroxynapthalene
Ankaflavin
Azanigerone
Betaenone
Burnettramic acid
Cercosporin
Cryptosporioptide
Epipyriculol
Flavoglaucin
Gregatin A
Harziphilone
Neosartorin
Scytalidin
Scytalone/T3HN
Secalonic acids
Terreic acid
UNII-YC2Q1O94PT
Yanuthone D
Monascorubrin
Tolypyridone C
Biscognienyne B
Abscisic acid
Number of identified secondary metabolites
0
1
2
3
4
5
